# Functional and ecological drivers of bacterial interactions in glacier-fed stream biofilms

**DOI:** 10.1128/msystems.00370-26

**Published:** 2026-06-02

**Authors:** Martina Gonzalez Mateu, Hannes Peter, Grégoire Michoud, David Touchette, Florian Baier, Nicola Deluigi, Richard Jacoby, Michael Zimmermann, Tom J. Battin

**Affiliations:** 1 River Ecosystems Laboratory, School of Architecture, Civil and Environmental Engineering, École Polytechnique Fédérale de Lausanne (EPFL) 27218, Lausanne, Switzerland; 2 Molecular Systems Biology Unit, European Molecular Biology Laboratory (EMBL), Meyerhofstrasse 1 9471, Heidelberg, Germany; University of Minnesota Twin Cities, St. Paul, Minnesota, USA

**Keywords:** bacterial interactions, biofilms, glacier-fed streams, metabolic cross-feeding, competition, coexistence

## Abstract

Biofilms are the dominant bacterial lifestyle, consisting of matrix-embedded, spatially structured communities where bacteria closely interact and coordinate their behaviors. Despite their ecological significance, the interplay among bacterial taxa that shapes biofilm community dynamics remains poorly characterized. We assessed the nature and drivers of interactions among bacterial strains isolated from a glacier-fed stream biofilm, using phenotyping, genomics, and metabolic fingerprinting. Biofilm bacterial coexistence was frequent (37% of pairs) even among strains with overlapping resource use and close phylogenetic relatedness. Positive interactions were evidenced through metabolic cross-feeding, synergy in biofilm formation, and interspecific biofilm induction. In competitive interactions, where one strain outcompeted the other, the dominant bacteria exhibited oligotrophic traits, such as higher carbon use efficiency, substrate specialization, high-yield strategies, and the production of antagonistic molecules. Our integrated approach demonstrates that efficient growth traits confer a competitive advantage, while widespread metabolic cooperation can facilitate bacterial coexistence. These findings provide critical insights into the forces shaping bacterial interactions and community assembly in stream biofilms.

**IMPORTANCE:**

Bacterial isolates have been extensively used across many systems to investigate how their interactions and traits shape coexistence and competition patterns. However, stream biofilm bacteria, despite forming the foundation of fluvial microbial ecosystems, have rarely been studied beyond their taxonomic composition, although deciphering their interactions is key to understanding ecosystem functioning. Here, we leveraged biofilm isolates from a glacier-fed stream to examine both competitive and positive interactions in co-culture, revealing the nature and key bacterial traits that mediate these interactions. By linking co-culture outcomes to microbial traits, this study uncovers the drivers of bacterial interactions in stream biofilm communities.

## INTRODUCTION

Microorganisms are social organisms. Complex intra- and inter-specific biotic interactions promote microbial diversity and underpin the success of microbial life (1, 2). As for other forms of life, microbial interactions span a wide range, ranging from symbiosis to competition. Evidence suggests that competitive interactions prevail in numerous microbial systems (3), including soils and plants (4–6), tree holes (7), and the gut microbiome of animals (8, 9), ranging from 39% to 89% of interactions. Resource scarcity, as is often the case in natural systems, supposedly fosters exploitative competition within microbial communities (10, 11). The prevalence of competitive interactions does not preclude the widespread occurrence of mutualistic interactions in microbial communities. Such positive interactions are often underpinned by metabolic exchanges (12, 13), which are, however, often difficult to infer from laboratory experiments (3, 14, 15). Nevertheless, some co-culture experiments have shown that isolates can coexist (16), cross-feed metabolites (17), and even over-yield compared to mono-species cultures (18).

Microbial biofilms are hallmarks of biotic interactions. The spatial organization of biofilms promotes niche diversification, which potentially reduces competition while promoting resource partitioning and metabolic cross-feeding at the same time (2, 19). The coexistence of different bacterial species can also facilitate the formation of biofilms as matrix production is often a cooperative process that is regulated by the secretion of specific compounds by various community members (20–22). The intrinsic relationships between spatial structure and positive interactions are thought to contribute to the success of microbial biofilms in numerous ecosystems (2, 23). This is certainly the case in extreme ecosystems, such as in hot springs and glacier-fed streams (GFS), where biofilms dominate life overall. In GFS, the biofilms that coat sediments host diverse and complex communities, including interactions across different kingdoms of life (24–26).

**Fig 1:**
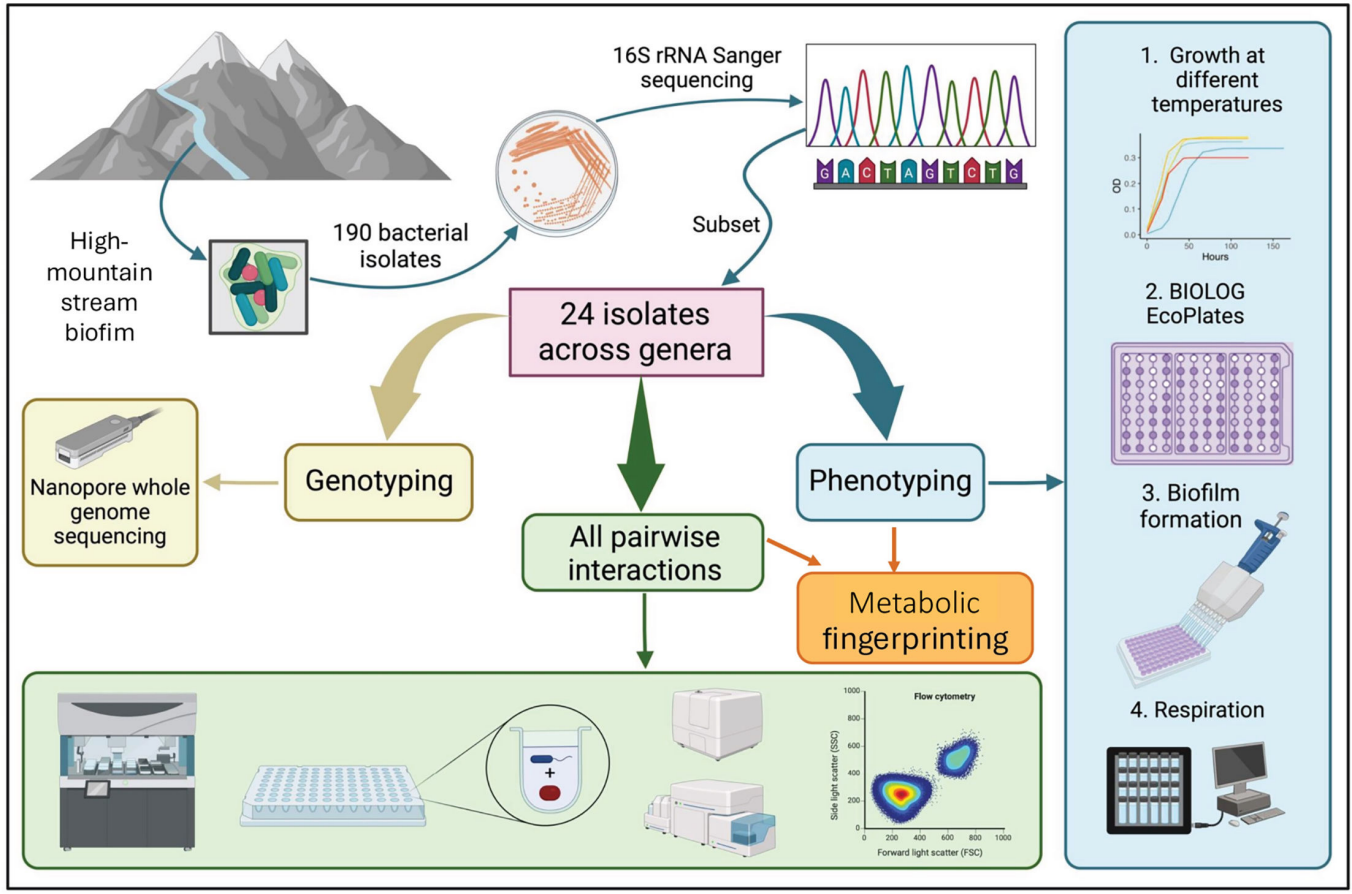
Depiction of the study’s workflow. Biofilms were grown on clay tiles in a glacier-fed stream in Val Ferret, Switzerland. Bacteria were isolated from these clay tiles in a variety of growth media, and 190 isolates were Sanger sequenced for taxonomic assignment based on the 16S rRNA gene. A subset of 24 isolates belonging to different genera and representative of common and rare taxa were selected for the study assays. Phenotypic characterization included testing the bacterial growth at different temperatures in ½ R2A media, the carbon substrate utilization based on Biolog Ecoplates, biofilm formation (in ½ R2A media and in conditioned media of other strains), and respiration. Genotypic characterization was done using MinION for whole-genome sequencing. Bacterial interactions were assessed by growing all isolates in monocultures and in all pairwise combinations (276 pairs), recording their absorbance at OD600 to obtain growth metrics, and evaluating the final composition of the pairs using flow cytometry and a random forest classifier. Metabolic fingerprinting was used to assess resource partitioning in ½ R2A media and cross-feeding interactions in conditioned media. Figure created using BioReneder.com.

Here, we assembled and characterized a culture collection of 24 phylogenetically and functionally diverse bacterial strains isolated from a GFS biofilm. Using this collection, we systematically tested all 276 possible pairwise co-cultures to determine whether cooperation or competition among taxa prevailed. Finally, we integrated genomic, phenotypic, and metabolic fingerprinting to uncover the traits driving these interactions (Fig. 1). We anticipated that due to the extreme conditions of GFS, these bacteria primarily engage in positive interactions in co-cultures, through cross-feeding, resource complementarity, and enhanced biofilm formation. Furthermore, we expected that phylogenetically distant pairs would use different resources, making coexistence more likely than for closely related pairs. Our multifaceted approach uncovered mechanisms and ecological trade-offs that shape bacterial interactions and likely contribute to the success of biofilms in GFSs.

## RESULTS

**Fig 2:**
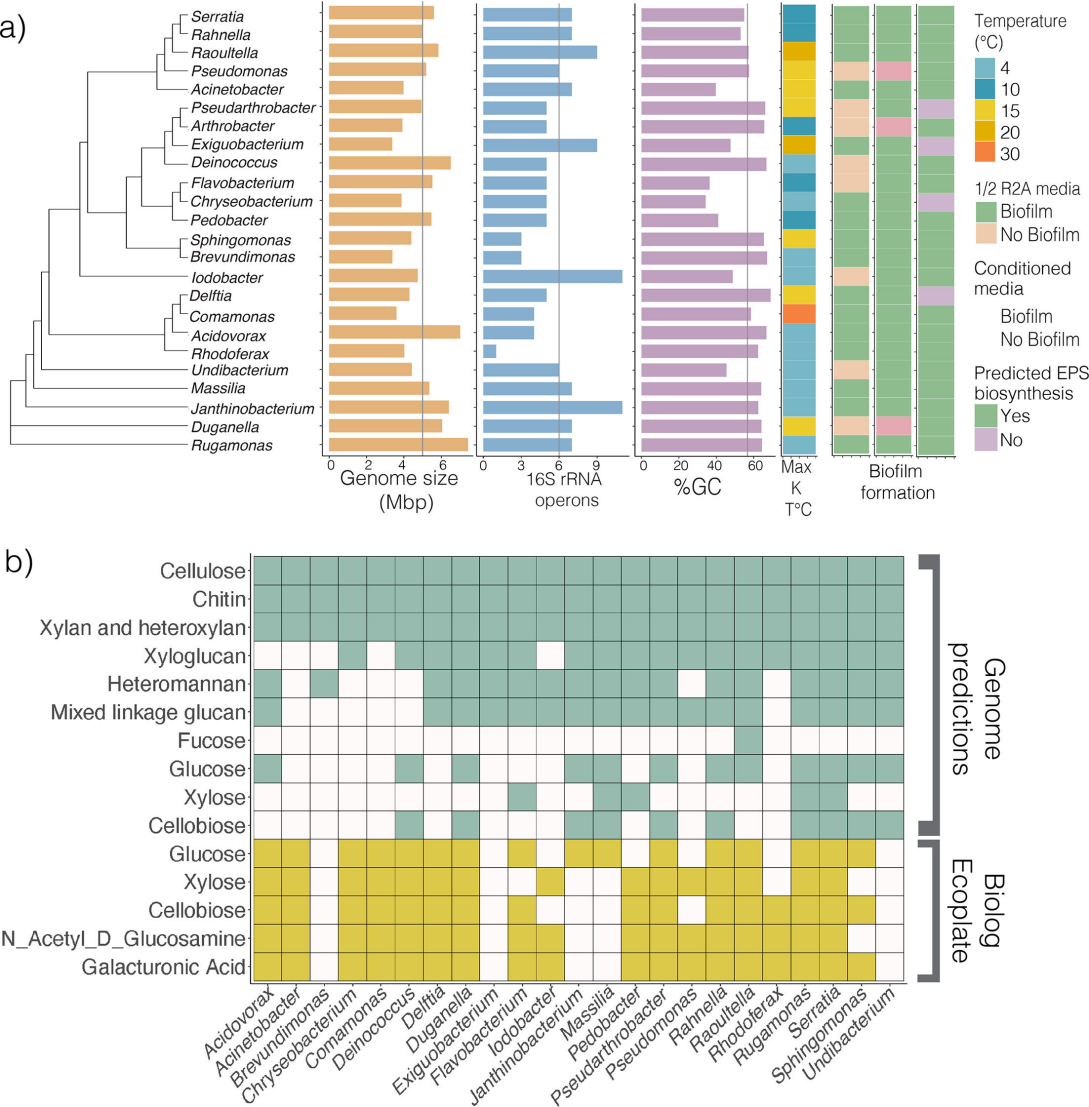
(**a**) Characterization of the 24 isolated strains in terms of their genome properties, temperature at which the isolates reached their highest carrying capacity (*K*), and biofilm-forming ability at 10°C, in conditioned media of at least one other strain, and as predicted based on genomic analysis (EPS = extracellular polymeric substances). The gray vertical lines indicate the mean values of each genome property for these strains. Taxonomy is shown at the genus level. (**b**) Presence/absence of degradation pathways of complex algal carbohydrates, and some of their byproducts (glucose, xylose, cellobiose, N-acetyl-glucosamine, and galacturonic acid). Genome-based classifications are shown as green tiles, indicating that the isolate had the genes required for substrate catabolism. The yellow tiles show the actual carbon substrate utilization based on the Biolog Ecoplates. Gray tiles indicate that the isolate either did not have the specific pathway for catabolism or was not able to utilize the carbon substrate in the laboratory.

We isolated and full-length sequenced the 16S rRNA gene of 190 bacterial strains, belonging to 43 genera, from GFS biofilms in the Dranse de Ferret (Valais, Switzerland) (Fig. 1). Our collection included members of the GFS core microbiome, such as Burkholderiales and Flavobacteriales, which are among the most prevalent in GFS worldwide (25; Fig. S1). From this collection, we retained for all experiments a non-redundant subset of 24 strains comprising both common and rare GFS bacteria (Fig. 2; Table S1).

### Phenotypic and genomic characterization

To characterize traits relevant to the ecology of GFS bacteria, we performed phenotypic and genomic analyses of the 24 strains (Fig. 2; Materials and Methods). Most strains were well-adapted to cold temperatures, reaching their highest carrying capacities (*K*) at 4°C–10°C, although a few (e.g., *Raoultella* and *Comamonas*) produced more biomass at higher temperatures (Fig. 2a). Average carbon use efficiency (CUE) across all strains was low (average ± SD: 0.13 ± 0.11; Table S1), consistent with the overall low CUE (median: 0.15) reported from GFS microbial communities worldwide (27). Not unexpectedly, 87% of the strains formed biofilms in laboratory assays (28), and even the few that did not encode the genomic potential for the biosynthesis of extracellular polymeric substances (EPS), the main component of the biofilm matrix. Whole-genome sequencing of the strains further revealed traits common in cryospheric bacteria (29, 30), including a high GC content (56% ± 10.7%) and high 16S rRNA operon copy numbers (6 ± 2.4; Fig. 2a) as previously predicted for GFS based on the rrnDB database, which provides information on rRNA operon copy numbers across bacterial genomes (31). Genome size of the strains was relatively constrained (5 ± 1.2 Mbp) and within the range typically found for environmental bacteria (32). Genomic predictions and Biolog Ecoplate assays showed that many strains metabolize or encode the genomic potential to metabolize algal-derived polymers (e.g., cellulose and xylan) and their degradation products (e.g., glucose, cellobiose, and xylose; Fig. 2b; Fig. S2), supporting earlier metagenomic evidence of algal–bacterial interactions in GFS biofilms (24, 26). Overall, these phenotypic and genomic traits reveal that the strains are well-suited to thrive in the cold and biofilm-dominated GFS ecosystems.

**Fig 3:**
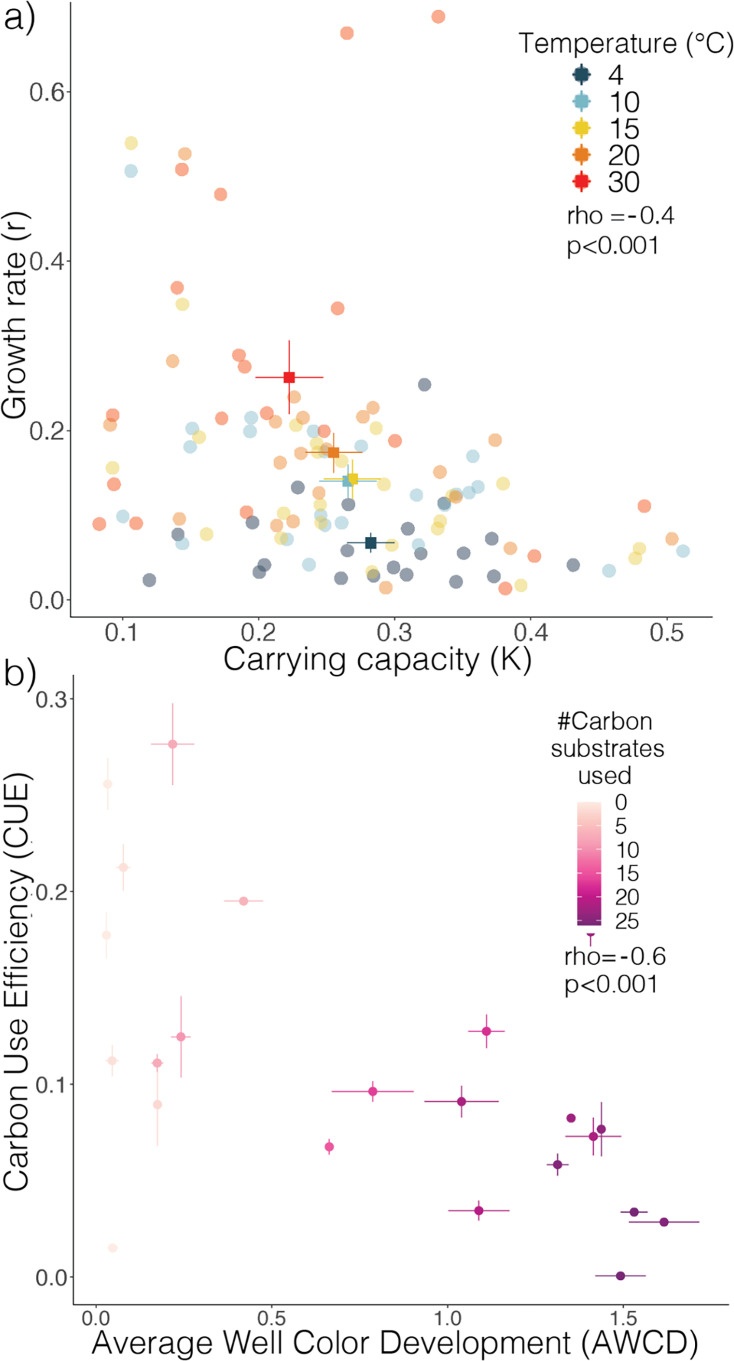
(**a**) Correlation between growth rate (*R*) and carrying capacity (*K*) of the 24 isolates across different temperatures. Darker squares show the mean and standard error for the 24 isolates for each temperature. (**b**) Correlation between carbon use efficiency and metabolic activity estimated as the average well color development (rho = −0.6, *P* < 0.001) and the number of carbon substrates utilized in the Biolog EcoPlates (shown in color gradient; rho = −0.57, *P* = 0.004). Error bars show the standard error of the mean.

Next, we examined the relationships between specific ecological traits that may influence bacterial interaction outcomes, including growth dynamics and carbon use efficiency (CUE) (Fig. 3; Materials and Methods). Growth rate (*r*), *K*, and CUE are key microbial traits that reflect distinct life history strategies and influence competitive ability (33, 34). We found that *r* and *K* were inversely related (Fig. 3a; rho = −0.4, *P* < 0.001), consistent with commonly observed trade-offs in bacteria between the ability to grow quickly and reaching high population densities (35, 36). This observed trade-off was further exacerbated by temperature, which increased *r* for most strains and lowered *K* (Fig. 3a). We also observed a trade-off between metabolic performance and efficiency. Carbon use efficiency declined with increasing metabolic activity, as inferred from Biolog Ecoplates (Fig. 3b; rho = −0.6, *P* < 0.001), reflecting the limited ability of strains with high CUE to use diverse carbon sources (Fig. 3b; rho = −0.57, *P* = 0.004). This supports the idea that investing in a broader range of pathways and enzymes to exploit diverse resources may increase respiratory and anabolic costs, ultimately reducing biomass production and growth efficiency, as previously suggested (37, 38). Together, these trade-offs reveal contrasting growth and metabolic strategies that may reflect different ecological strategies of bacteria in GFS biofilms.

### Interaction patterns

To explore how variation in bacterial traits and phylogeny influences interaction among the 24 isolates, we systematically tested all 276 possible pairwise co-cultures. Pairwise interaction outcomes were evaluated by training a random forest classifier on flow cytometry data of monocultures and subsequently applying the classifier to flow cytometry data of co-cultures (Materials and Methods). Interactions were classified as coexistence when the final abundance of both strains ranged between 25% and 75%, whereas dominance by one strain (≥75% abundance) was classified as competition. We used the Pianka index to assess the relationship between interaction outcomes and potential niche overlap between strains (index values near one indicate a high niche overlap). We expected that variation in ecological traits and phylogeny would help explain which strains coexist and which outcompete others.

**Fig 4:**
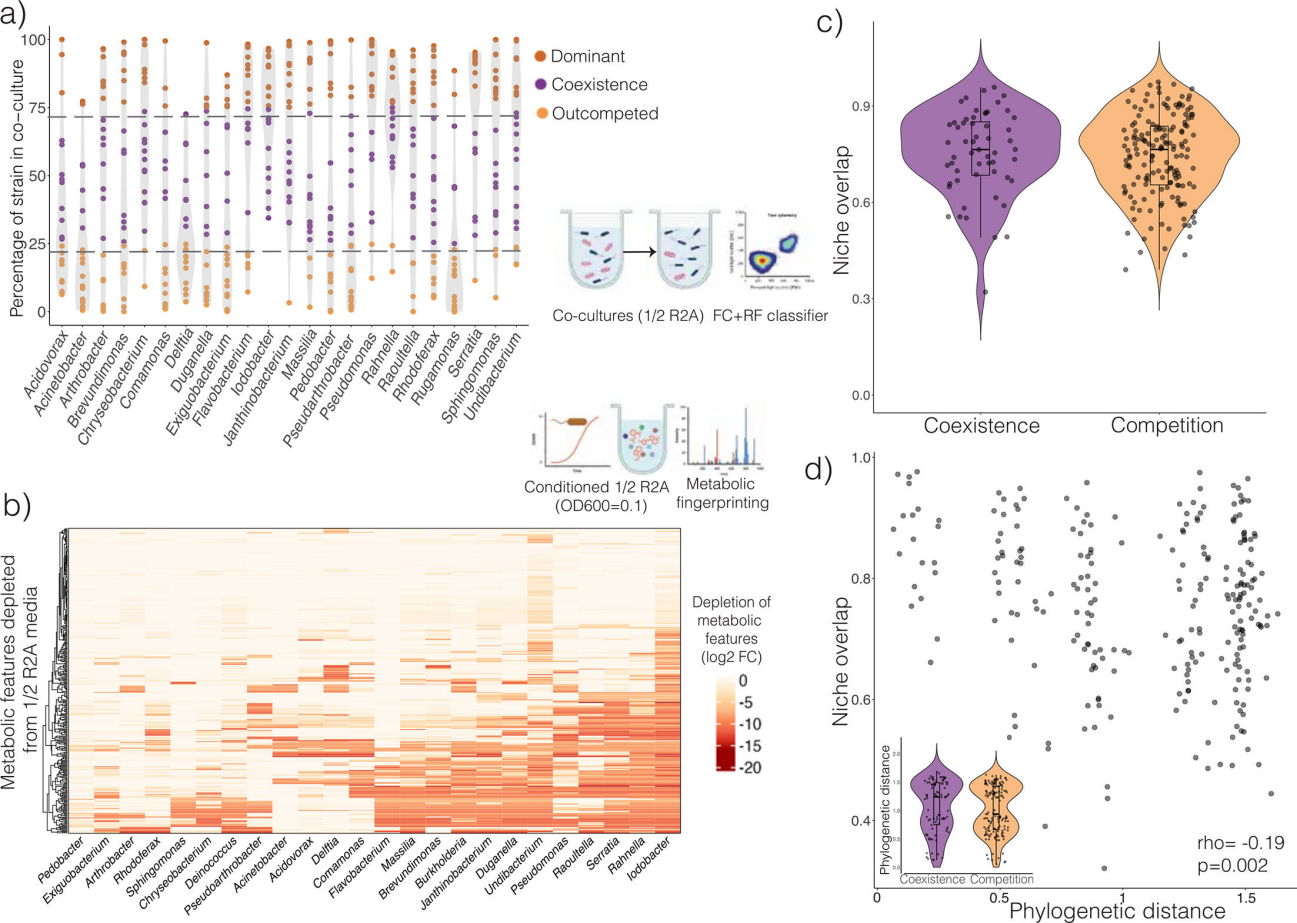
Interaction outcomes across pairwise experiments in ½ R2A growth media (**a**) plotted for each strain. Interaction outcomes were determined by flow cytometry (FC) paired with a random forest classifier (RF). When one of the two isolates had >75% abundance at the end of the experiment, it was considered to be dominant, while abundances of <25% indicate it was outcompeted. Coexistence occurred when both isolates were found in abundances between 25% and 75% (**b**) Heatmap of metabolic features depleted by each strain in ½ R2A shown as log2 fold change. (**c**) Violin and boxplot showing interaction outcomes based on niche overlap of pair members. (**d**) Correlation between the phylogenetic distance and niche overlap (Pianka index) of interacting pairs. The inset shows the interaction outcomes (coexistence and competition based on phylogenetic distance, *P* > 0.05).

Coexistence was observed in 37% of the 242 pairwise interactions that the random forest classifier could confidently differentiate based on flow cytometry data (Fig. 4a). Relatively high instances of coexistence remained frequent even when using different stringency thresholds to classify the interactions: with a 65%/35% cutoff, 20.6% of pairs were still classified as coexisting, whereas a less stringent 80%/20% cutoff classified 50% of pairs as coexisting. Based on these results, the selected 75% cutoff provided a conservative estimate for classifying coexistence (Fig. S3), avoiding both overly permissive (≥80%) and overly restrictive (≤60%) classifications. To better understand this pattern, we examined whether phenotypic differences (i.e., differences in *K*, *r*, CUE, and carbon substrate use between interacting pairs), often linked to microbial coexistence (39, 40), influenced interaction outcomes. Interestingly, greater phenotypic differences did not promote coexistence (Table S2). Instead, pairs composed of two specialists or two generalists, categorized according to their breadth of carbon substrate utilization in Biolog Ecoplate assays, were more likely to coexist (Table S2, *P* = 0.02). We expected the coexistence in co-cultures could be a result of resource partitioning (41, 42). However, metabolic feature analysis of growth media utilization revealed that most strains preferentially utilized similar substrates that led to high niche overlap among coexisting pairs, as estimated using the Pianka index (mean Pianka index of 0.75 and 0.74 for coexisting and competitive pairs, respectively; Fig. 4b and c). Coexistence was also expected to be common among phylogenetically distant bacteria that tend to have lower niche overlap (10). Consistent with this expectation, niche overlap was negatively correlated with phylogenetic distance, although some distant strains still showed high overlap (rho = −0.19, *P* = 0.002; Fig. 4d). Nevertheless, coexistence was common among both phylogenetically close and distant pairs (Fig. 4d). These findings indicate that coexistence in GFS bacteria may not be driven by trait differences and can arise even among phylogenetically similar bacteria with overlapping resource use, suggesting that additional mechanisms contribute to their coexistence.

**Fig 5:**
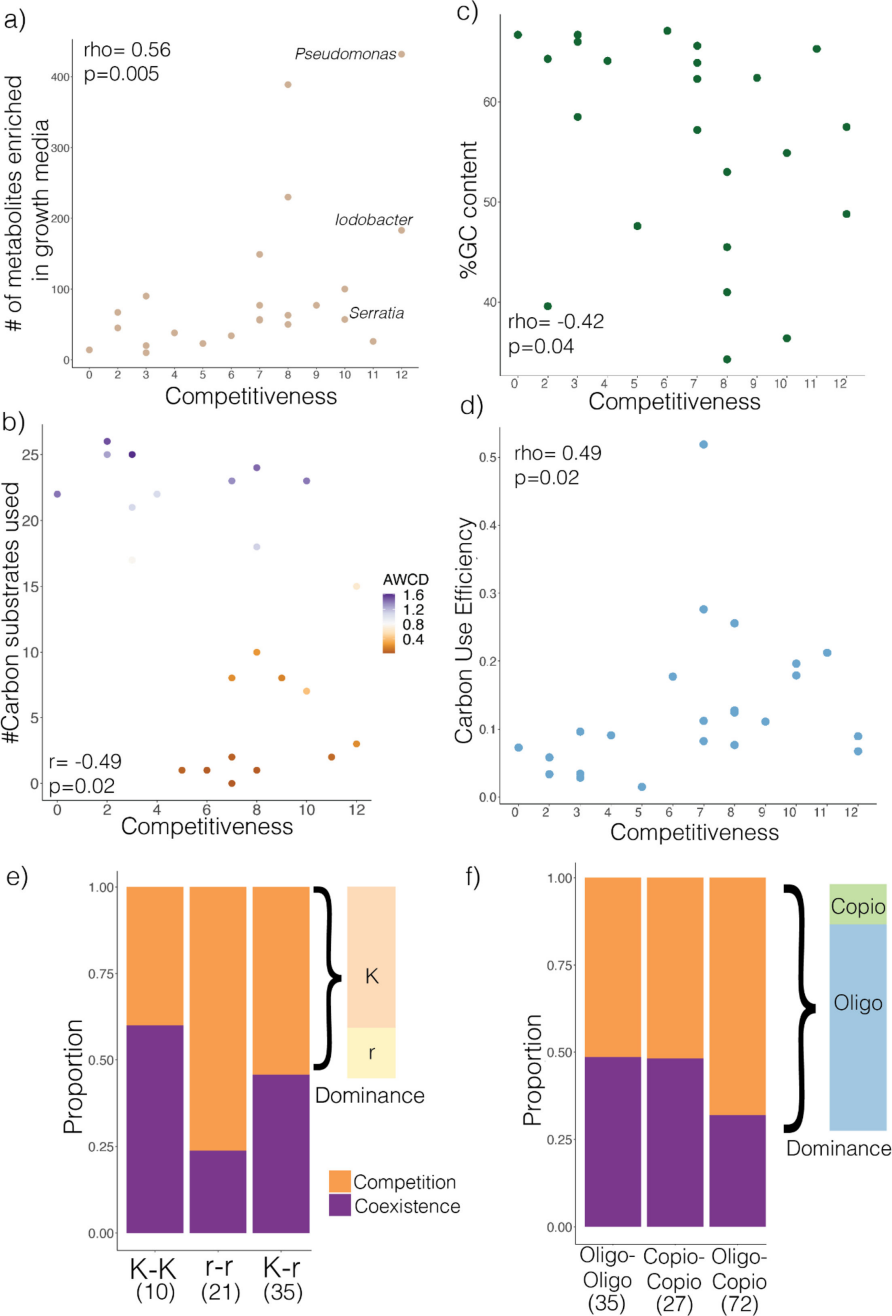
Correlation between the strain’s competitive ability (defined as the number of other strains that an isolate competitively excluded) and (**a**) the number of metabolic features significantly enriched by each strain growing in ½ R2A media, (**b**) the number of carbon substrates the strains utilized in the EcoPlate assay, colored by isolate’s metabolic activity, (**c**) isolate’s %GC content, and (**d**) carbon use efficiency. (**e and f**) Bar plots showing the proportion of pairwise interactions in which isolates with similar or contrasting life history strategies either competed or coexisted. The sidebar illustrates the proportion of cases where (**e**) *K*-selected, *r*-selected, or (**d**) oligotrophic, copiotrophic strains dominated (abundance >75% in co-culture) in competitive interactions between strains with contrasting ecological strategies (*K-r* and Oligo-Copio). Numbers in parentheses show the number of pairs in each category.

Most pairwise interactions (63%) were competitive, resulting in one strain dominating numerically by the end of the incubation. To better understand the nature of competitive interactions in co-culture, we leveraged phenotypic, genotypic, and metabolic fingerprinting data to explore traits enabling certain strains to outcompete others (Materials and Methods). We defined each strain’s competitive ranking as the number of other strains it was able to outcompete in co-culture. For instance, *Iodobacter*, *Sphingomonas*, and *Flavobacterium* were among the most competitive, outcompeting 52%, 48%, and 44% of other strains, respectively. Strains with higher competitive ability were associated with a greater number of metabolic features detected in the growth media (Fig. 5a; rho = 0.56, *P* = 0.005). For example, *Iodobacter* and *Pseudomonas*, the most competitive strains (Table S1; Fig. 5a), produced 389 and 432 metabolic features, respectively, whereas *Acidovorax* and *Delftia*, among the weakest competitors, produced only 10 and 14 features, respectively. The release of diverse compounds, including antibiotics, toxins, or waste products, may serve as a strategy to inhibit competitors (43, 44). For example, the deeper analysis of specific metabolic features via MS/MS fragmentation revealed that *Iodobacter* and *Serratia* released siderophores (serratiochelin and vanchrobactin, respectively; Fig. S4 and S5) that can directly mediate bacterial competition by sequestering iron from competitors (43, 45). Therefore, the ability to chemically modify the environment through metabolite release may represent an important competitive trait in these GFS biofilm bacteria.

We also found that traits linked to efficient growth were potential drivers of competitive success. Specifically, bacteria with greater substrate-use specialization, relatively lower %GC content, and higher CUE more frequently outcompeted other strains in co-cultures (Fig. 5b, c, and d). These traits are often characteristic of oligotrophic bacteria (46–48), suggesting that efficient growth could be advantageous in nutrient-poor GFS. Regarding metabolic efficiency, the competitive advantage was specifically linked to the maximization of biomass per unit of carbon processed (CUE) rather than the minimization of respiration, as specific respiratory efficiency did not correlate with competitive ranking (rho = 0.2, *P* = 0.3). Interestingly, some of the most competitive strains, which had elevated CUE and resource specialization, belonged to groups, such as *Flavobacterium, Rhodoferax,* and *Sphingomonas*, that are abundant in GFS (Table S1, Fig. S6).

We then explored how competitive traits, including efficient growth and resource specialization, correspond to broader ecological strategies and how these strategies influence coexistence and competition patterns (Materials and Methods; Fig. S7). Classification of strains as *r*-strategists (fast growers with low *K*) versus *K*-strategists (slow growers with high *K*) and as oligotrophs (metabolically efficient resource specialists) versus copiotrophs (less efficient resource generalists) allowed us to identify bacteria with contrasting life-history strategies within our data set. Surprisingly, we found that outcomes of bacterial interactions were not influenced by growth and trophic strategies of the pairs (i.e., *r* vs*. K*, oligotrophs vs. copiotrophs; logistic regression, *P* > 0.05; Fig. 5). Although not significant, bacteria with high-yield (i.e., high *K*) strategies displayed relatively high instances of coexistence (60% in *K-K* pairs relative to 45% in *r-K*, and 23% in *r-r*; Fig. 5e), while *r-r* pairs exhibited higher competition (logistic regression, *P* = 0.057; Fig. 5e). However, when strains with contrasting strategies were paired and the interaction was competitive, the dominant strains were predominantly *K*-strategists (χ² = 4.3, *P* = 0.03) accounting for 74% of pairs. Similarly, dominant strains were predominantly oligotrophs (χ² =22.2, *P* < 0.001), accounting for 84% of competing pairs (Fig. 5e and f). Combined, these results reiterate that efficient growth, characteristic of *K*-strategists and oligotrophs, resulted in a competitive advantage. Although absolute CUE values are low in GFS compared to other ecosystems, our data show that even modest increases in resource-use efficiency can provide a competitive advantage in these nutrient- and carbon-poor environments.

### Cross-feeding and enhanced biofilm formation

**Fig 6:**
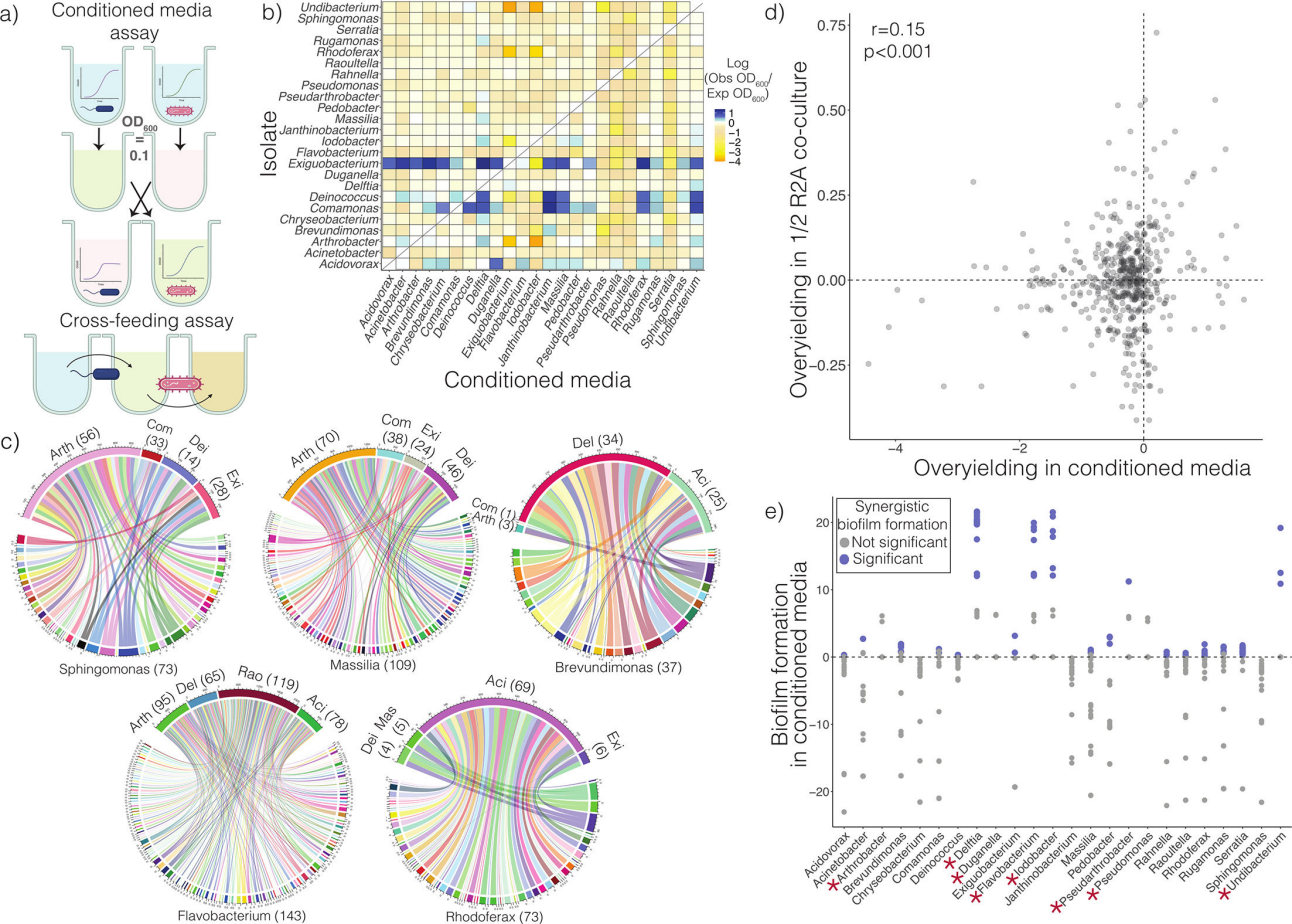
(**a**) Setup of conditioned media assay and cross-feeding assay. (**b**) Heatmap showing over/underyielding as the yield (OD600) of each strain (*y*-axis) growing in the conditioned media of other strains (*x*-axis) relative to their own yield in ½ R2A (Expected OD600). Blue tones show overyielding, while yellow/orange tones show underyielding. (**c**) Chord diagrams showing metabolic cross-feeding between common glacier-fed stream genera (donors, bottom) and overyielding recipients (top) in conditioned media. Numbers in parentheses indicate metabolic features enriched by donors and depleted by recipients. Abbreviations: Arth = *Arthobacter*, Aci = *Acidovorax*, Com = *Comamonas*, Dei = *Deinococcus*, Del = *Delftia*, Exi = *Exiguobacterium*, Rao = *Raoultella*. (**d**) Correlation between overyielding of strains growing in conditioned media and in ½ R2A co-cultures. Overyielding in the *y*-axis is quantified as the Log of the carrying capacity (*K*) of co-cultures in ½ R2A divided by the *K* of the highest yielding monoculture for pairs and in the *x*-axis as the Log of the observed *K* in conditioned media divided by the *K* in ½ R2A. (**e**) Biofilm formation of each strain in conditioned media of other strains is expressed as the log of biofilm formation in conditioned media (Observed OD595) relative to their biofilm formation in ½ R2A at 10°C (Expected OD595). Blue points represent instances of significantly greater biofilm formation in conditioned media of other strains (14% of cases in which the 95% CI of the observed mean was above the 0 line).

Given the high frequency of coexistence observed, we expected that facilitative interactions such as cross-feeding could be common. We therefore assessed the potential for cross-feeding by growing each strain in media previously conditioned by every other strain (49), in combination with untargeted metabolic fingerprinting to track potential resource exchanges (Materials and Methods; Fig. 6a). We found that in 30% of cases, strain yields in conditioned media were not significantly different from their yields in ½ R2A, while in 13% of instances strains significantly over-yielded in conditioned compared to fresh media (Fig. 6B; Fig. S8). Thus, although 57% of strains showed reduced growth in conditioned media, likely due to a reduction in nutrients or potentially inhibition, it is striking that in 43% of cases strains grew equally well or better despite nutrient depletion. While nutrient depletion and metabolic facilitation can be confounded in conditioned media assays, the frequent observation of equal or greater growth relative to the fresh medium is unlikely to be explained by depletion alone, given prior resource consumption and the high overlap in resource use among strains (Fig. 4b). We expected that efficient oligotrophic taxa, which are common in GFS, would benefit the most from metabolic byproducts, as they generally invest in a broad array of transporters (31). However, we found that strains as *Exiguobacterium*, *Comamonas*, and *Deinococcus,* which are generally rare in GFS biofilms, exhibited low *K* values (~0.1; Table S1) in ½ R2A but achieved higher yields (reaching up to 242% ± 8.4%, 295% ± 10.4%, and 181% ± 34.6% greater OD_600_, respectively) in conditioned media of other strains (Fig. 6b; Fig. S8a). This conditioned media assay therefore suggests that facilitative interactions are common and likely related to metabolic cross-feeding. Indeed, metabolic fingerprinting supported this cross-feeding hypothesis by confirming that these rare GFS strains utilized metabolites secreted in the conditioned media of more abundant strains such as *Massilia*, *Rhodoferax*, *Brevundimonas*, *Flavobacterium*, and *Sphingomonas* (Fig. 6c; Table S1). By linking the utilization of secreted metabolites to instances of improved growth, we confirmed that biochemical facilitation is likely a key driver of the observed over-yielding in the conditioned media assay. Moreover, we investigated whether higher yields in conditioned media were associated with overyielding in ½ R2A co-cultures, a phenomenon observed in 38% of co-cultured pairs (Fig. S8b). We expected that strains that showed improved growth in conditioned media would also be more likely to show overyielding in ½ R2A media when grown with specific partners, possibly due to the establishment of cross-feeding interactions. Indeed, we found a significant positive correlation between improved growth in conditioned media and higher yields in the ½ R2A co-culture assay (Fig. 6d; *r* = 0.15, *P* < 0.001). Although OD_600_ may not capture absolute biomass due to morphological diversity, our assessment of how specific strains responded to different conditioned media relative to their own monoculture baselines allowed for a reliable quantification of growth shifts. Overall, these findings suggest that cross-feeding can be a key interaction that promotes growth and coexistence among bacteria in GFS biofilms.

In addition to metabolic cross-feeding, positive interactions between strains were evident in their ability to engage in synergistic biofilm formation. A biofilm assay (28) evaluating the effects of conditioned media on biofilm formation showed that 14% of the co-cultures displayed synergistic biofilm formation, defined as significantly higher biofilm formation of a strain in conditioned medium of another compared to its biofilm formation in fresh ½ R2A medium (Fig. 6e; Fig. S9a). Although the proportion may seem small, it is notable because these assays used conditioned media, suggesting that some strains release metabolites that actively facilitate biofilm formation in others despite nutrient depletion. Interestingly, five of the eight strains that failed to form biofilm as monocultures in fresh ½ R2A, such as *Flavobacterium* and *Undibacterium*, did form biofilms when grown in a conditioned medium of certain strains (Fig. 2a and 6e), consistent with interspecific biofilm induction. Our results highlight the role of secreted compounds in mediating cooperative behaviors that enhance biofilm formation, highlighting how chemical signals can shape microbial community interactions in GFS biofilms.

## DISCUSSION

Bacteria engage in a wide spectrum of positive and negative interactions, but the sign and strength of these interactions depend on resource availability and other environmental cues (8, 50). Exploitative competition (i.e., negative interactions) typically prevails among bacteria that have similar metabolic requirements for scarce resources (10, 51). Spatially organized biofilms, however, may relax competition through niche differentiation and effective sharing of metabolites (2, 19, 52). Our finding of relatively frequent bacterial coexistence (37% of the pairwise interactions), even among strains with overlapping resource use and close phylogenetic relatedness, supports this notion.

The frequency of coexistence in our study is higher than typically reported for microbial systems (e.g., <19% [3, 14]), while in other studies, when high coexistence is observed, it is usually in host-associated systems (53, 54). The observed coexistence is striking, given the nature of our experimental environments, which limit niche diversity and select for heterotrophic, relatively fast-growing, and primarily aerobic bacteria. Microbial coexistence that rests on facilitative interactions and metabolic cross-feeding may contribute to the success of the biofilm mode of life in GFS. In fact, it has been suggested that metabolic interactions and the efficient recycling of nitrogen in GFS biofilms are advantageous in these ultra-oligotrophic ecosystems (24).

We anticipated coexistence to occur primarily among strains with different ecological traits (i.e., lower niche overlap) or greater phylogenetic distance, which is often associated with metabolic dissimilarity (10, 55). For instance, trade-offs illustrated by inverse relationships between *r* and *K,* or CUE and metabolic breadth, would be indicative of promoting coexistence by enabling complementary ecological strategies (36, 39, 40). Indeed, a negative relationship between *r* and *K* is thought to be a hallmark of cooperative behavior in biofilm communities, potentially contributing to bacterial coexistence, as strains with different growth strategies may utilize resources differently and occupy complementary ecological niches (41, 52). However, contrary to expectations based on previous empirical studies (18, 55, 56), we found that coexistence was common among strains with similar traits, elevated niche overlap, and close phylogenetic relatedness, suggesting that differences in traits and phylogeny alone do not determine interaction outcomes and that other mechanisms may be involved. For instance, strains may exhibit fine-scale differences in metabolic strategies by utilizing low-abundance substrates differently (57, 58), display fitness differences in the use of shared substrates that enable coexistence (59), or, as supported by our findings, engage in facilitative interactions such as metabolic cross-feeding.

Indeed, the 37% coexistence rate we observed is mirrored by the 43% frequency of strains that grew well in conditioned media, suggesting that facilitative interactions likely contribute to coexistence outcomes. We frequently observed cross-feeding among strains, supporting its relevance for bacterial growth in complex communities. In oligotrophic GFS, cross-feeding could be an important mechanism for nutrient recycling and maximizing resource utilization, as observed in marine systems (60), allowing diverse taxa to coexist. We found that the growth of many strains (e.g., *Arthrobacter, Acidovorax,* and *Comamonas*) was stimulated in conditioned media, and metabolic fingerprinting revealed that strains classified as rare in GFS (e.g., *Deinococcus, Delftia, Arthrobacter,* and *Exiguobacterium*; Table S1) even benefited from specific metabolic byproducts from prevalent community members. This is consistent with findings from plant and soil microbial communities (12), where metabolic byproducts from dominant taxa supported low-abundance species, suggesting that cross-feeding may similarly help maintain diversity in GFS communities. The spatial proximity of bacteria in biofilms likely facilitates metabolic cooperation and enhances the exchange of public goods (19, 61), which is particularly important for auxotrophs (14). Our experiments did not include auxotrophic strains, which are common in natural systems (62), and may therefore underestimate the occurrence of metabolic cross-feeding in GFS biofilms. Therefore, it is likely that metabolic cooperation is even more widespread in natural GFS biofilms.

Harsh environments and fluctuating nutrient availability are thought to foster positive interactions among bacteria (16). While our culture medium was relatively nutrient-poor, it did not capture the temporal variability or pronounced nutrient limitations characteristic of GFS. In addition, the use of static batch cultures resulted in the accumulation of metabolic byproducts that altered the chemical environment compared to a continuous flow system. While this metabolite accumulation certainly does not capture the natural nutrient fluxes in GFS, it can reflect the chemical gradients and microenvironments within natural biofilms (63). Overall, our experimental setup likely favored the detection of cross-feeding interactions while still reflecting the localized chemical conditions experienced by cells in natural GFS biofilms.

Our study also uncovered the significance of positive interactions for biofilm formation, which constitutes a key adaptive trait of microbial life in streams (61, 63). This is supported by the fact that 87% of the strains formed biofilms in the laboratory, and those that did not still encoded the genomic potential for the synthesis of EPS, a fundamental constituent of biofilms. Biofilm formation results from cooperative efforts among bacteria (2, 64), and prior coexistence can influence bacterial behavior, promoting such cooperation (2, 21, 65). Consistent with this, we observed interspecific biofilm induction in otherwise non-biofilm-forming strains when grown in conditioned media of other specific strains. Our study further revealed multiple instances of synergistic biofilm formation, as has been similarly observed among strains obtained from other systems (e.g., marine, soil, and food contact surfaces) (21, 66, 67). Interestingly, these synergistic interactions occurred without physical contact between strains, which is often considered key for multi-species biofilm formation in streams (68) and other environments, including oral biofilms (69), soils (70), and marine algal surfaces (66). Although nutrient depletion (71) or stress (72, 73) could also promote microbial biofilm formation, the strain-specific responses we observed both for intraspecific biofilm induction and overall synergistic biofilm formation suggest that EPS production was more likely related to metabolic exchanges, including, for example, quorum-sensing molecules or EPS precursors released by specific strains, and not a general outcome of starvation or other stress imposed by conditioned media. Overall, these results highlight that chemical cues can drive mixed-strain biofilm formation, emphasizing the role of these signals in supporting cooperative interactions in GFS.

Besides positive interactions, our experiments also revealed competitive interactions related to the growth of certain strains. In line with expectations for *r*- and *K*-specialists, we found that high-yielding and oligotrophic strains (e.g., *Flavobacterium* and *Rhodoferax*) were better competitors than fast-growing and copiotrophic strains (e.g., *Exiguobacterium* and *Acinetobacter*). The latter often dominate in experimental co-cultures with well-mixed conditions (74, 75). Although ½ R2A is a complex medium, it has relatively low nutrient concentrations, and therefore, we expected oligotrophic and *K*-strategists to outcompete copiotrophs or *r*-strategists (76, 77) as was observed. Our results are consistent with modeling studies showing that resource-efficient and slow-growing bacteria can outcompete faster-growing bacteria in spatially structured environments and when engaged in metabolic cross-feeding (52, 78). Notably, some of the most prevalent genera found in GFS worldwide, including *Rhodoferax*, *Flavobacterium*, *Sphingomonas*, and *Brevundimonas* (25), were among the highly efficient oligotrophs in our study, which aligns with the expectation that efficient resource use is a key selective pressure in GFS biofilms.

Our metabolic fingerprinting analyses further revealed that the competitive success of these strains was linked to a greater number of metabolic features secretion. For example, strains with higher competitive ability, such as *Iodobacter* and *Pseudomonas*, released a greater diversity of metabolites and created some of the most inhibitory conditioned media for other strains (Fig. 6b). While most of these features remain unannotated, the positive correlation between metabolic feature richness and competitive ranking suggests that the secretion of a diverse array of metabolites may increase the likelihood of modifying the chemical environment or producing specific antagonistic molecules. A chemically diverse exometabolome can influence competitors through multiple mechanisms, including the release of inhibitory compounds, modification of the chemical conditions, and alteration of resource availability within the biofilm matrix (79). In line with this, we identified two types of siderophores that are important drivers of social interactions in natural environments (50) and can directly inhibit competitor growth by sequestering available iron (43, 45). These molecules exemplify how secreted metabolites can mediate competition by limiting access to micronutrients. Together, our results highlight that both resource-use efficiency and the ability to modify the chemical environment contribute to competitive success in these stream biofilm communities.

Our study reveals that bacterial interactions in GFS biofilms are shaped by a balance of metabolic cooperation and competitive efficiency. We show that metabolic exchanges can support bacterial growth and biofilm formation and can foster coexistence even among ecologically similar taxa. In contrast, efficient growth and the ability to modify the environment emerged as key determinants of competitive success. By linking specific microbial traits to both cooperative and competitive outcomes, our study provides new insights into the fundamental processes underlying microbial interactions in stream biofilms.

## MATERIALS AND METHODS

### Experimental setup

Bacterial isolates were obtained from biofilms grown on clay tiles in a glacier-fed stream at 1,774 m above sea level in Val Ferret, Switzerland (45.906 N, 7.124 E) (73). The stream is part of the Dranse de Ferret catchment, which spans 20.24 km^2^ and has 1.78% glacier coverage, influencing stream dynamics through glacier melt (72; https://metalp.epfl.ch, last accessed: 26 April 2025). Biofilms were scraped off the tiles, suspended in sterile stream water, and bacteria were streaked for isolation onto various types of media (R2A, ^1^/_10_ R2A, ^1^/_50_ R2A, streamwater agar media, nutrient agar, and LB agar). Plates were incubated either at room temperature or at 4°C until colonies were visible for re-streaking for further isolation. A culture collection was obtained, and isolates were stored in either R2A, LB, or diluted R2A broth, based on their original isolation media, with 10%–20% glycerol at −80°C. Over 400 bacteria were isolated, and 190 of these were selected for Sanger sequencing based on morphological differences or haphazardly when colonies were too small. Individual colonies were picked, lysed using GeneReleaser (BioVentures Inc.), and the 16S rRNA gene was PCR-amplified using the primer set 27F and 1492R (80). The PCR products were submitted for sequencing to StarSEQ (Mainz, Germany) or Microsynth AG (Balgach, Switzerland).

For the different assays, we selected 24 strains from our culture collection ensuring to include representatives from genera that are commonly found in glacier-fed streams (like *Rhodoferax*, *Massilia*, *Acidovorax*, *Flavobacteria*, *Brevundimonas*, and *Sphingomonas*) (24, 25, 81) and randomly selecting others that are rare community members (Fig. S1, Table S1).

### Long-read whole genome sequencing of bacterial strains

To sequence the genomes of the 24 strains, fresh cultures of 5 mL R2A medium in 14 mL culture tubes were inoculated from individual colonies grown on R2A agar plates at room temperature. Cultures were shaken at 300 rpm at room temperature for 2 days before harvesting the cells at 10,000 × *g* for 10 min at 4°C. Pellets were kept frozen at −20°C until DNA extraction with Zymo Quick-DNA Fungal/Bacterial Miniprep Kit (Zymo Research, Cat. No. D6005) following the manufacturer’s protocol. DNA concentrations and purity were determined using the Qubit dsDNA HS kit with a Qubit fluorometer (Thermo Fisher) and NanoDrop One Spectrophotometer (Thermo Fisher), respectively. DNA size distribution and integrity were determined using an Agilent 2200 TapeStation system with genomic DNA ScreenTape (Agilent Technologies). DNA concentrations measured with Qubit were in the range of 60–120 ng/µL with 50 µL eluent and their DNA integrity numbers ranged from 6.2 to 8 for TapeStation analysis.

Libraries for long-read Oxford Nanopore Sequencing were prepared with the Rapid Barcoding Kit 24 V14 (Oxford Nanopore Technologies, Cat. No. SQK-RBK114.24) according to the protocol and normalized for sequencing to 400 ng of DNA per strain/barcode on R.10.4 flow cells in a MinION Mk1B (Oxford Nanopore Technologies) device using the MinKNOW v23.11.5 software with high-accuracy base calling. Two library preparations and sequencing runs were performed to compensate for a lower read depth of several barcodes during the first run.

### Carbon substrate use

BIOLOG Ecoplates (Biolog Inc.) were used to characterize the carbon substrate utilization profiles and overall metabolic activity of the strains. Bacteria were grown in 15 mL culture tubes with R2A broth for 48 h at room temperature, centrifuged, and washed free of growth medium with 1× PBS before normalizing the cultures to an OD_600_ of 0.2. The Ecoplates were inoculated with 100 µL of each culture in triplicate, incubated at 10°C, and the absorbance at OD_590_ was measured over 2 weeks until color development stabilized.

### Growth at different temperatures

To measure growth parameters across different temperatures, cultures were initially grown for 48 h at each experimental temperature and normalized to an OD_600_ of 0.02 using ½ R2A in 96-well plates. Blank controls containing ½ R2A were included along the edges of all plates, which were incubated at 4°C, 10°C, 15°C, 20°C, and 30°C. Plates were shaken for 30 s prior to each measurement, and absorbance at OD_600_ was measured at least twice a day until the strains reached the stationary phase.

### Carbon use efficiency (CUE)

Carbon use efficiency of bacterial monocultures grown at 10°C was estimated as BCP/(BCP + *R*), where BCP is the bacterial carbon production during the exponential growth phase and *R* is the respiration rate.

Flow cytometer data of each strain (methods below) were used to obtain the cell densities at the end of the log phase and to estimate bacterial cell sizes. Polystyrene size standard beads with known particle size (Spherotech, Cat. No. NPPS-4K) were run in parallel on the flow cytometer to estimate bacterial cell size. The carbon content of bacterial cells was calculated based on cell volume (82) across three replicates per strain. Bacterial carbon production was then computed using a linear regression fit to temporal changes in bacterial carbon content.

Respiration rates were measured using four PreSens SDR SensorDish sets (PreSens, Germany), each with four 24-well plate sensors (total 96) and 5 mL sensor glass vials. Strains were grown for 48 h at room temperature, with shaking (Heidolph Titramax 1,000 at 900 rpm) and another 24 h at 10°C (no shaking) for temperature adaptation in 1 mL R2A media in a 96 deep-well plate (Fisher Scientific AG, 12597927) covered with a membrane foil (Sigma, Z763624-100EA). Pre-cultures were briefly shaken and normalized to an OD_600_ of 0.15 in 900 µL pre-chilled (10°C) ½ R2A using a Tecan Fluent pipetting robot. Sensor glass vials were filled with 4.8 mL ½ R2A and pre-chilled to 10°C. From each culture, 250 µL were used to inoculate three replicate sensor glass vials and immediately placed on the PreSense SDR SensorDish at 10°C and measured every 2 min for 8 h. The sensor unit was beforehand calibrated to 10°C measurements using fully oxygenated and oxygen depleted (1% sodium sulfite) water as described in the manual. The linear slopes of oxygen depletion from 60 min to 150 min of measurement were used to calculate respiration rates.

### Pairwise interactions

To investigate pairwise interactions, the 24 bacteria were grown in monoculture and in all possible pairwise combinations (276 unique pairs) using 96-well plates. All mono and pairwise combinations were assayed in triplicate, starting from three independent colonies on agar plates to inoculate single strain cultures. Strains were grown for 48 h at 10°C in ½ strength R2A, normalized to an OD_600_ of 0.02, and inoculated into 180 µL of the same media in 96-well plates using a Tecan Fluent liquid handling robot. Using custom scripting for the Tecan robot, replicates of all pairwise combinations were randomly assigned on the plates to avoid any location bias. For monocultures, 20 µL of the culture was added, while for the 276 pairwise combinations, 10 µL of each culture was added, resulting in a substitutive design; all edges of the plate were used as blank controls and filled with the medium. Plates were incubated under stationary conditions at 10°C, and the readings of absorbance at OD_600_ (Biotek Synergy H1) were taken at least twice a day for 5 consecutive days, with 30 s of shaking prior to each measurement.

### Flow cytometry for cell abundance

To measure the relative abundance of each strain in the pairwise combinations at the end of the 5-day incubation at 10°C, we analyzed all mono and pairwise cultures with flow cytometry. For this, 1 µL of each well was transferred in a U-bottom 96-well plate containing 198 µL of 3.7% of filtered formaldehyde (Sigma, Cat. No. 252549) in PBS buffer and kept at 4°C until analysis. Before flow cytometry analysis, 2 µL of 50 µM SYTO-13 (Life Technologies, Cat No. S7575, 0.5 µM final concentration) were added to each well, shaken for 30 s, and incubated for 20 min in the dark. Plates were subsequently run in the flow cytometer (ACEA NovoCyte 1000 and NovoSampler Combo) using an acquisition speed of 14 µL min^−1^ with a core diameter of 7.7 µm. Events with a signal larger than 1,000 in the SYTO-13 fluorescence channel and forward scatter larger than 100 were recorded, and the stop conditions were set to either 20,000 gated events or 2 min.

### Conditioned medium assay and biofilm formation

A conditioned medium assay was set up by growing each culture in the conditioned ½ R2A media of all others. To obtain the conditioned media, the 24 cultures were grown from single colonies in 50 mL Falcon tubes with ½ R2A at 10°C for 48–72 h until they reached an OD ~0.1; this is about half of the median expected OD_600_ for these strains (median = 0.24). Falcon tubes were centrifuged at 5,000 × *g* for 10 min to pellet the cells, and the supernatant was removed and filter-sterilized using a 0.22 µm filter into a new tube. The sterile conditioned media were then dispensed into 96-well plates. Another set of cultures was set up in 2 mL 96-well plates and grown in ½ R2A for 48 h to serve as the inoculum. The OD_600_ values of these cultures were normalized to 0.02, and 20 µL was added into wells containing 180 µL of each of the conditioned media, resulting in 576 combinations that were assessed in triplicate. Positive controls consisted of all cultures growing in regular ½ R2A, and negative controls consisted of uninoculated conditioned media of each strain; all controls were also assessed in triplicate. Plates were incubated at 10°C for 5 days, and OD_600_ values were recorded daily.

After the experiment, biofilm formation was evaluated by staining with 0.01% crystal violet (28). Briefly, the 96-well plates were washed three times with DI water, stained with 205 µL of 0.01% crystal violet, shaken for 20 min, and rinsed again with DI water. The crystal violet was then solubilized with 210 µL of 70% ethanol, incubated for 30 min on a shaker, and the absorbance was read at OD_595_.

### Metabolic fingerprinting

To investigate metabolic signatures of consumption and secretion, we undertook various liquid chromatography coupled to mass spectrometry (LC-MS) measurements involving conditioned media. First was an experiment designed to determine the metabolic fingerprint of each strain upon the growth medium, where five replicates of each strain (50 µL of normalized culture at 0.02 OD_600_) and five replicates of sterile ½ R2A controls were incubated in 450 µL of ½ R2A medium at 10°C until the cultures reached an OD600 of ~0.1, as described in the previous assay. Cultures were then centrifuged and stored at −80°C. In a separate experiment designed to investigate cross-feeding, we prepared an additional 96-well plate containing four replicates of specific strains (*Sphingomonas, Flavobacterium, Rhodoferax, Brevundimonas,* and *Massilia*), and four sterile ½ R2A controls and incubated under the same conditions to create conditioned media for the cross-feeding assay. Once the cultures reached the OD_600_ of ~0.1, the media were centrifuged and filtered (2 µm) to remove the cells. These sterile conditioned media were subsequently used to grow a subset of strains that had previously exhibited enhanced growth in the conditioned media from these donor strains, indicating potential cross-feeding interactions. The experiment was set up by inoculating 20 µL of each OD_600_-normalized strain into 180 µL of the corresponding conditioned medium and growing them at 10°C for 60 h. Sterile controls included four replicates of ½ R2A and sterile conditioned media of the five donor strains, enabling the subsequent analysis of feature consumption and secretion through LC-MS. Samples were centrifuged and stored at −80°C for LC-MS analyses.

To prepare samples for untargeted LC-MS, 20 µL of the culture supernatant were extracted by the addition of 80 µL mixture of acetonitrile:methanol (1:1) and then incubated at −20°C for 1 h and centrifuged at 4,500 × *g* for 15 min at 4°C to remove insoluble material. For untargeted detection of more polar metabolites, a hydrophilic interaction liquid chromatography (HILIC) chromatography approach was employed according to the method of (PMID: 34497420). This involved drying 20 µL of extracted metabolites via vacuum centrifuge, and resuspending the sample in 5 µL of resuspension solution (50% acetonitrile, 5 mM ammonium acetate, pH 9.0). Next, 3 µL of the sample were injected onto an HPLC (Agilent Infinity 1290) and separated via a Poroshell 120 HILIC-Z column (150 × 2.1 mm, 2.7 µm particle size). Solvent A was 5 mM ammonium acetate in water plus 0.5% deactivator additive (Agilent), pH 9.0; solvent B was 5 mM ammonium acetate in 90:10 acetonitrile water plus 0.5% deactivator additive (Agilent), pH 9.0. Chromatographic gradient was 4% solvent A for 2 min, increasing to 12% solvent A until 5.5 min with a hold until 8.5 min, then increasing to 14% solvent A at 9 min with hold until 14 min, then increasing to 18% solvent A at 17 min, then increasing to 35% solvent A at 23 min with hold until 24 min, then reequilibration at 4% solvent A until 32 min. HPLC flow was ionized using electrospray ionization at 225°C, and mass spectra were acquired over the mass range 50–1,700 using a Q-TOF (Agilent 6550) operating in negative ionization mode with a scan rate of 1.5 spectra/s calibrated to reference masses 112.9855 and 1,033.9881. In a separate untargeted analysis aimed at detecting apolar metabolites, including secreted antimicrobial compounds, 10 µL of extracted metabolites were mixed with 10 µL of water, and 15 µL were injected onto an HPLC (Agilent Infinity 1290) and separated via a Poroshell HPH C18 column (100 × 2.1 mm, 1.9 µm particle size). Solvent A was 0.1% formic acid in water, and solvent B was 0.1% formic acid in methanol according to the method of (PMID: 39085612). Chromatographic gradient was 5% solvent B for 0.1 min, increasing to 95% solvent B at 5.5 min with hold until 6.5 min, followed by re-equilibration at 5% solvent B until 8 min. HPLC flow was ionized using electrospray ionization at 275°C, and mass spectra over the mass range 50–1,700 Da were acquired using a Q-TOF (Agilent 6550) operating in a positive ionization mode with a scan rate of 1.5 spectra/s calibrated to reference masses 121.0508 and 922.0097. In addition, we conducted follow-up measurements designed to collect MS/MS fragmentation spectra for a small subset of MS features in specific samples of particular interest. For these measurements, we focused our efforts on the C18 data set, due to higher peak intensities and better chromatographic performance compared to HILIC. However, even in the C18 data set, this was a challenging task, with manual inspection revealing that only six features of interest gave high-quality mass spectra (MS2 base peak intensity >1,000 counts). The experimental procedure involved re-injecting selected samples using identical chromatographic and ionization parameters, then collecting CID MS/MS spectra for a predefined set of RT-mz features, using manually constructed inclusion lists specific for each sample. Specifically, MS1 ions present in the inclusion list and detected in the MS1 scan with intensity >1,000 counts, RT ± 0.5 min, and mz ± 100 ppm were selected for MS/MS fragmentation in the subsequent scan, with a precursor isolation width of 1.3 Da. Collision energy was chosen by manual inspection of derived spectra acquired with default CE of 20 eV and then refined with subsequent measurement at higher or lower CE values where necessary.

### Data analysis

All data analysis and figure plotting were carried out in RStudio (version 4.2) and using ggplot2 (Wickham, 2016).

### Phenotypic data analysis

Growth parameters (*r* and *K*) of monocultures and pairwise combinations were obtained from fitting a logistic growth model using the GrowthCurver R package (v.0.3.1) (83). Specifically, the intrinsic population growth rate, *r*, and the maximum size that the population can reach in the growth environment, K (carrying capacity), were estimated from the logistic equation: $N_{t}=K/\left(1+\left(\left(K-N_{0}\right)/N_{0}\right)\times e^{\left(-rt\right)}\right)$, where *N*_*t*_ describes the population size at time *t* and *N*_0_ is the population size at the beginning of the growth curve. Two of the pairs were excluded from the analysis because of poor model fit; hence, the analysis included 274 pairs.

We used Biolog EcoPlates to assess carbon substrate utilization and evaluate the differences in metabolic profiles between strains. The OD_590_ of the Ecoplates was blank-corrected, and the average well color development (AWCD), which is an indicator of metabolic activity, was calculated as the sum of the blank-corrected OD_590_ divided by the total number of substrates. The use of specific carbon substrates was defined as positive when the blank-corrected OD_590_ absorbance values were greater than 0.2 (84) for at least two of the three replicates. The strain *Arthrobacter* was removed from the analysis because it did not grow in the Biolog Ecoplate media.

Strains were considered capable of biofilm formation when the measured absorbance values were higher than three standard deviations above the mean OD_590_ of the blank controls (sterile ½ R2A media) for at least two of the three replicates. Blank-corrected absorbance values were used to estimate synergistic biofilm formation in conditioned media as the log-ratio between the carrying capacity of a strain in conditioned media and the carrying capacity observed in ½ R2A. The mean and 95% confidence intervals were used to define the instances of significant synergistic biofilm formation.

### Genomic data analysis

Sanger forward and reverse sequences were assembled, quality-trimmed (Geneious Prime 2022.1.1), and classified using the BLAST NCBI 16S rRNA database. Accession numbers for the culture collection are available in the NCBI GenBank (accession numbers: PQ796245PQ796429).

Long reads from the Oxford Nanopore sequencing were filtered using Filtlong (v0.2.1) (https://github.com/rrwick/Filtlong) and assembled using Flye (v2.9.3) (85) or Raven (v1.8.3) (86); assemblies were then polished using Medaka (v1.11.3) (https://github.com/nanoporetech/medaka), and seqkit (v.2.7) (87) was used to get statistics on the assemblies. The best assembly was selected using checkm2 (v1.0.1) (88), and the genomes were annotated using bakta (v.1.9.2) (89) and eggnog-mapper (v2.1.13) (90). The genome taxonomy was further confirmed using GTDB-Tk (v2.5.2) (91). Phylogenetic distances based on the whole genome data were estimated using the cophenetic.dist function in the ape R package (v.5.8-1) (92). Genome sequences are available in NCBI GenBank under the BioProject ID PRJNA1171349 (accession numbers in Table S1).

The microTrait pipeline (93) was used to identify ecologically relevant traits related to resource use, biofilm formation, and energy generation pathways in our genomes. GapMind (94) was then used to find the most likely pathway for the catabolism of specific carbon compounds. For comparison with our laboratory Biolog Ecoplate assays, the strains were considered potentially capable of utilizing carbon sources when all the expected steps of a specific catabolic pathway were found in the genome (“high confidence” in the Gapmind prediction).

### Pairwise interaction outcomes

Interaction outcomes were assessed at the end of the incubation period, when strains were in the stationary phase (Fig. S10) (7, 75), to reflect how species coexist or exclude one another under resource limitation and metabolic byproduct accumulation, as might be observed within natural biofilms. Random forest models were used for the classification of cells in pairwise cultures based on flow cytometry measurements. For this, all optical parameters recorded by the flow cytometer (i.e., height and area of forward- and side-scatter as well as three fluorescence wavelengths [530 nm, 572 nm, and 675 nm]) of both monocultures and pairwise cultures were log-transformed and z-scaled. For each pairwise culture, a random forest classifier as implemented in the randomForest R package (4.7-1.2) (95) was trained based on 5,000 randomly selected flow cytometry measurements from both constituent monocultures. Specifically, area-integrated signal of forward (FSC) and side-scatter (SSC) of the 488 nm laser, as well as fluorescence signals in 530 nm, 572 nm, and 675 nm emissions (488 nm excitation) were used to train the classifiers. To evaluate classifier performance, the monoculture data sets were split 80:20 into train and test data sets, and the out of bag (oob) error was used to estimate model accuracy for each pair. To evaluate classified performance, the monoculture data sets were split 80:20 into train and test data sets, and the oob error was used to estimate model accuracy for each culture pair. Finally, the random forest model was used to differentiate the cells in pairwise cultures. The strain *Deinococcus* exhibited very low events in monoculture, and we were unable to classify it in the pairwise co-cultures; hence, pairs that included *Deinococcus* were excluded from the analysis. The average model accuracy was 0.95 with a 95% CI ranging from 0.942 to 0.959. Pairs with less than 80% oob accuracy (*n* = 11) were excluded. This cutoff corresponds to Cohen’s Kappa values < 0.6. Therefore, only strain pairs for which the model distinguished cell populations substantially better than by chance were kept, resulting in 220 pairs analyzed.

Pairwise interactions were classified as competitive if the percentage of a strain was >75% at the end of the experiment or as coexistence if the abundance of each strain ranged between 25% and 75% based on the flow cytometer data and random forest classification. Competitive ranks were assigned to each strain based on these interaction outcomes, as the number of instances in which each strain was able to competitively exclude another; higher ranking strains were better competitors and had a higher number of interactions that resulted in competitive exclusion. To see if pairwise interaction outcomes related to phylogenetic distances and to identify significantly overyielding strains, we used a Wilcoxon signed-rank two-sided test.

To evaluate the effects of life history strategies (*r* vs. *K*, and oligotroph vs. copiotrophs) and genotypic traits (genome size, copies of 16S, and %GC) on interaction outcomes, we used logistic regression using a binomial generalized linear model. The model was fitted using the glm function with a logit link function.

Overyielding in pairwise combinations was assessed as the log(observed/expected) value for carrying capacity (*K*), estimated from OD_600_-based growth curves; the expected value of *K* was defined on a pairwise basis as that of the highest yielding monoculture within each pair. To assess whether pairs were significantly over or under yielding, we used 95% confidence intervals of the means; if the uncertainty associated with a pair crossed the 0 line, it was considered not significant (i.e., the growth in pairs did not differ from the growth observed for the most productive monoculture). Similarly, in the conditioned media assay, overyielding was defined in terms of the carrying capacity observed when the strains grew in conditioned media and the expected carrying capacity, which corresponded to the growth of that strain in ½ R2A. Since OD_600_ measurements can be affected by strain morphology (e.g., different cell sizes, shapes, or aggregation properties), we used these relative *K* metrics to ensure that assessments of overyielding were based on within-strain comparisons across treatments. By normalizing growth in conditioned media to each strain’s baseline performance in the same fresh medium, we control for strain-specific optical properties and focus on relative performance changes across treatments, making OD_600_ a suitable metric for relative growth responses under these conditions. The Wilcoxon rank-sum test was used to assess if overyielding differed between coexistent and competing pairs and between pairs that either exhibited or did not exhibit synergistic biofilm formation.

Correlation analyses were used to assess the relationship between competition outcomes, phenotypic and genotypic parameters, and Bonferroni corrections for multiple testing were used when performing multiple comparisons.

### LC-MS data analysis

The rationale of our LC-MS data analysis strategy was to define the metabolic fingerprints of strains in the culture medium. This involved identifying metabolic features that were (i) reproducible across the 5× replicates and (ii) significantly released or depleted between strains versus the matched sterile controls. However, we did not attempt to systematically annotate the molecular identity of the thousands of detected MS features, primarily because the experimental setup used R2A medium, which is a complex and undefined chemical mixture containing yeast extract, peptone, and casamino acids (96, 97), and therefore, a compositional analysis of this medium was outside the scope of the study. Procedurally, acquired LC-MS spectra were first converted from .D to .MZML using MSConvert (98) using generic default settings. Next, untargeted feature extraction and alignment were conducted using MZmine 3. Briefly, the mass detection module was set up with a noise filter of 500 counts. The ADAP chromatogram builder module was used to construct EICs for masses that occurred in five consecutive scans, each with a minimum intensity of 1,000 counts, and at least one scan where intensity was over 2,000 counts, with *m*/*z* tolerance set to either 3.7 mDa or 15 ppm. To align peaks between samples, the join aligner module was used, with RT tolerance of 0.1 min for C18 and 0.5 min for HILIC, *m*/*z* tolerance of either 3.7 mDa or 15 ppm, weight for *m*/*z* 3, and weight for RT 1. The feature list rows filter module was used to filter out MS-features detected in two or fewer samples.

The derived LC-MS data were analyzed using the MetaboDiff R package (v. 0.9.5) (99) to identify differentially abundant metabolic features. This involved two parallel strategies to define features which were either (i) utilized or (ii) secreted by the strains relative to sterile control. To estimate resource utilization by each strain growing in ½ R2A, we assessed the metabolic features significantly depleted by strains versus a sterile control based on the HILIC data set, which had an initial size of 751 features. The HILIC data set was chosen because the methodology is well-suited to detecting metabolites utilized as bacterial growth substrates. Pre-processing steps included removing any feature not present in at least four of the five ½ R2A sterile control replicates, which left 510 MS features. Next, the mean abundance of any depleted feature had to be less than 50% of the mean abundance of the five ½ R2A controls, which left 379 features. Finally, the remaining features were subject to a *t*-test between strain versus sterile control using the diff_test function with Benjamini-Hochberg *P*-value correction for multiple testing (*P* < 0.05), which left 340 MS-features categorized as utilized for further analysis. From this list, resource utilization of each strain on ½ R2A was then characterized using the EcoSimR package (v. 0.1.0) (100), and niche overlap was calculated based on the Pianka index (101). This metric is used to quantify the similarity in resource use between different organisms based on the proportional utilization of shared resources. The index was calculated as follows: $O_{j}k=O_{k}j=\left(\sum \left(p_{i}j\times p_{i}k\right)\right)/\left(\sqrt{\left(\sum {\left(p_{i}j\right)}^{2}\sum {\left(p_{i}k\right)}^{2}\right)}\right)$, where $p_{ij}$ and $p_{ik}$ represent the proportional use of resource i by strains j and k, respectively, based on the 340 MS features. The resulting values range between 0 and 1 and indicate exclusive resource use or overlapping resource utilization, respectively.

To define metabolic features that were secreted by the strains, we used the C18 data set that had an initial size of 4,080 features. Again, the C18 data set was chosen because this methodology is well-suited to detecting antimicrobial compounds. Pre-processing steps involved removing all features which were detected in the sterile controls (mean EIC intensity > 6,000 counts), which left 737 features. Next, metabolic features were filtered to remove any feature detected in fewer than four replicates of any single strain, which left 731 features. Finally, the remaining features were subjected to a *t*-test between strain versus sterile control, and differentially abundant features were identified using the diff_test function with Benjamini-Hochberg *P*-value correction for multiple testing (*P* < 0.05), which left 656 MS-features categorized as secreted for further analysis.

For compound identification via MS/MS, individual MS2 spectra were manually selected and exported to .MGF using Agilent Qualitative Analysis software, and imported into SIRIUS version 6 (102). The SIRIUS module was used for molecular formula identification, and the CSI:FingerID module was used for structural database search. Mass accuracy thresholds for MS2 peaks were set to 10 ppm, allowed elements were set to H, C, N, O, P, and S, and all databases were searched against (PubChem, BioCyc, ChEBI, COCONUT, DSSTox, GNPS, HMDB, KEGG, KNApSAcK, LOTUS, LipidMaps, MeSH, NORMAN, SuperNatural, and YMDB). This workflow had a relatively low success rate, with only two of the MS/MS spectra being successfully matched to a database entry via the CSI:FingerID module.

On the other hand, cross-feeding interactions were investigated on the HILIC negative data set by first identifying features secreted by specific strains relative to ½ R2A sterile controls. These features were then assessed for depletion by other strains when grown in the conditioned media of the first strain (reflecting the metabolic products released by the first strain), compared to sterile conditioned media controls. Cross-feeding interactions were visualized using the circlize package (v. 0.4.16) (103).

## Data Availability

Genome sequences are available in NCBI GenBank under the BioProject ID PRJNA1171349. Accession numbers for the culture collection are available in the NCBI GenBank (accession numbers: PQ796245–PQ796429). Data used in this study and code to recreate the figures can be found in Zenodo (https://doi.org/10.5281/zenodo.17339143).

## SUPPLEMENTAL MATERIAL

Supplemental material msystems.00370-26-s0001.pdf Tables S1 and S2 and Figures S1 to S10.

ASM does not own the copyrights to Supplemental Material that may be linked to, or accessed through, an article. The authors have granted ASM a non-exclusive, world-wide license to publish the Supplemental Material files. Please contact the corresponding author directly for reuse.
