# Supplementary material for: Functional and ecological drivers of bacterial interactions in glacier-fed stream biofilms
Source: mSystems. 2026 Jun 2;11(6):e00370-26. doi: 10.1128/msystems.00370-26 (PMC13288933; doi:10.1128/msystems.00370-26)
Supplement: Supplemental material — Tables S1 and S2 and Figures S1 to S10. [file msystems.00370-26-s0001.pdf]

Supplementary Table 1: Genotypic and phenotypic characteristics and NCBI accession numbers. Abundance is based on the source community of the isolates (Touchette et al., 2025)[1]. CUE=carbon use efficiency, AWCD= Average Well Color Development, K= carrying capacity, r= Growth rate. Competitive ability reflects the number of other bacteria that each strain copmetitively excluded in pariwise interactions.

Strain	% Abundance of Genera	Genome completeness	Genome size (Mbp)	Genome Contamination	16S rRNA	%GC	CUE	Respiration ($mgCO_2$-C $L^{-1}h^{-1}$)	AWCD	K (10°C)	r (10°C)	Competitive Ability	GenBank Accession number
Acidovorax sp	0.45	87.7	7.01	2.91	4	66.7	0.03	11.38	1.62	0.24	0.04	3	JBRIJX000000000
Acinetobacter bohemicus	1.13	99.5	3.97	5.39	7	39.6	0.03	3.73	1.51	0.19	0.20	2	JBRIJW000000000
Arthrobacter sp.	<0.01	95.3	3.92	1.47	5	65.6	0.52	0.69	0.02	0.51	0.06	7	JBRIJV000000000
Brevundimonas bullata	0.55	85.4	3.37	3.97	3	67.1	0.18	4.10	0.03	0.22	0.07	6	JBRIJD000000000
Chryseobacterium piscicola	0.05	100.0	3.86	0.23	5	34.3	0.08	3.20	1.44	0.32	0.12	8	JBRIJU000000000
Comamonas sp.	0.01	94.8	3.60	3.49	4	58.5	0.10	0.63	0.79	0.10	0.10	3	JBRIJT000000000
Deinococcus sp.	<0.01	100.0	4.29	0.15	5	69	0.001	5.20	1.47	0.14	0.07	NA	JBRIJS000000000
Delftia acidovorans	<0.01	93.7	6.50	1.48	5	66.7	0.07	1.85	1.41	0.25	0.10	1	JBRIJS000000000
Duganella sp.	<0.01	100.0	6.05	0.67	7	64.1	0.09	3.26	1.06	0.15	0.18	4	JBRIJR000000000
Exiguobacterium undae	<0.01	94.3	3.36	1.94	9	47.6	0.02	2.27	0.05	0.11	0.51	5	JBRIJQ000000000
Flavobacterium sp.	2.92	86.2	5.52	2.17	5	36.4	0.20	2.98	0.42	0.28	0.18	10	CP199950
Iodobacter sp.	<0.01	100.0	4.74	0.9	11	48.8	0.09	2.81	0.21	0.26	0.09	12	JBRIJP000000000
Janthinobacterium sp.	<0.01	89.9	6.40	1.51	11	62.4	0.11	8.59	0.17	0.35	0.13	9	JBRIJO000000000
Massilia sp.	0.08	100.0	5.35	0.25	7	63.9	0.11	3.38	0.05	0.32	0.07	7	JBRIJN000000000
Pedobacter sp.	<0.01	92.8	5.47	4.02	5	41	0.12	4.95	0.24	0.34	0.12	8	JBRIJM000000000
Psuedarthrobacter sp.	<0.01	81.5	4.93	1.15	5	66	0.03	3.53	1.09	0.46	0.03	3	JBRIJL000000000
Pseudomonas sp.	0.64	91.6	5.19	2.28	6	57.5	0.07	14.83	0.65	0.36	0.13	12	JBRIJK000000000
Rahnella inusitata	<0.01	87.0	4.99	6.11	7	53	0.13	5.159	1.11	0.25	0.17	8	JBRIJJ000000000

Raoultella terrigena	<0.01	100.0	5.84	0.83	9	57.2	0.08	4.10	1.35	0.34	0.11	7	JBRIJI000000000
Rhodoferax sp.	10.43	92.3	4.01	1.98	1	62.3	0.28	2.75	0.22	0.15	0.20	7	JBRIJH000000000
Rugamonas sp.	0.09	98.9	7.44	4.22	7	64.3	0.06	2.48	1.28	0.25	0.09	2	CP199949
Serratia proteamaculans	<0.01	91.8	5.61	3.81	7	54.9	0.18	1.97	1.41	0.36	0.17	10	JBRIJG000000000
Sphingomonas sp.	1.57	75.6	4.39	2.86	3	65.3	0.21	3.75	0.08	0.11	0.64	11	JBRIJF000000000
Undibacterium sp.	0.05	92.5	4.42	1.67	6	45.5	0.26	1.33	0.03	0.19	0.21	8	JBRIJE000000000

[1]Touchette, D., Mateu, M. G., Michoud, G., Deluigi, N., Marasco, R., Daffonchio, D., Peter, H., & Battin, T. (2025). Experimental evidence on the impact of climate-induced hydrological and thermal variations on glacier-fed stream biofilms. *FEMS Microbiology Ecology*, *101*(1), fiae163. https://doi.org/10.1093/femsec/fiae163

Supplementary Table 2: Logistic regression results of interaction outcomes (Coexistence and Competition) and phenotypic differences in isolate co-cultured pairs.

	Estimate	Std. error	Z value	Pr(>|z)
Intercept	0.009864	0.302734	0.033	0.9740
Differences in r	-0.237927	0.933449	0.255	0.7988
Differences in K	-0.526170	1.734391	0.303	0.7616
Differences in CUE	0.149276	1.363529	0.109	0.9128
Differences in carbon substrate use	-0.040365	0.017378	-2.323	**0.0202**

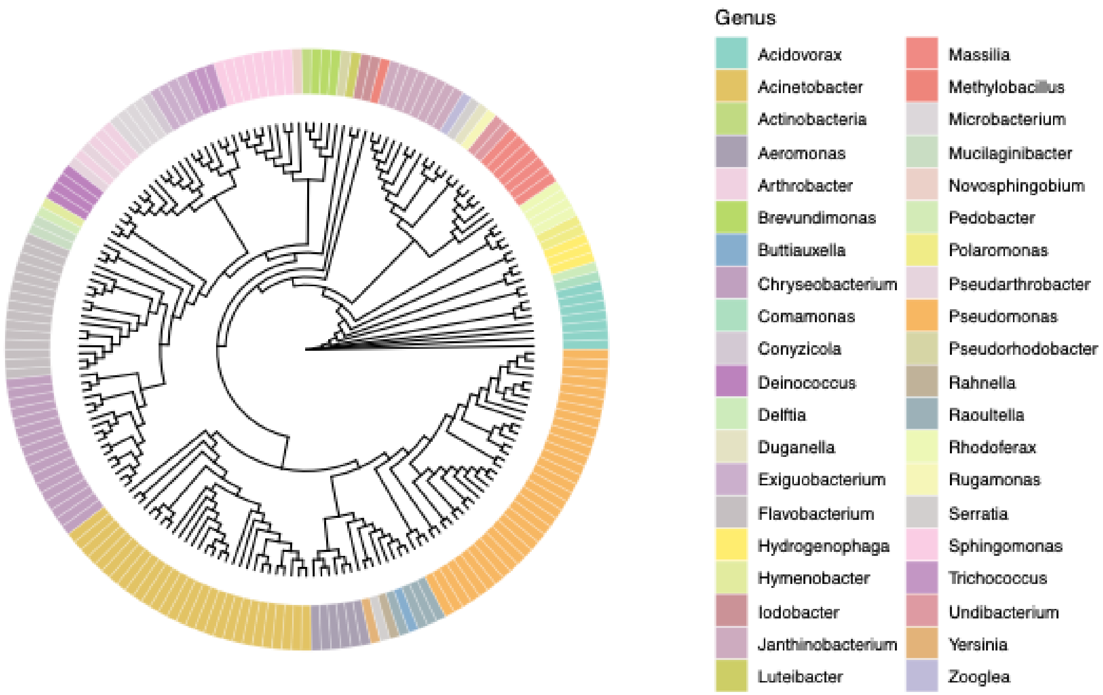

Supplementary Fig. 1: Phylogenetic tree showing the Genera of 190 bacterial strains from a glacier-fed stream culture collection.

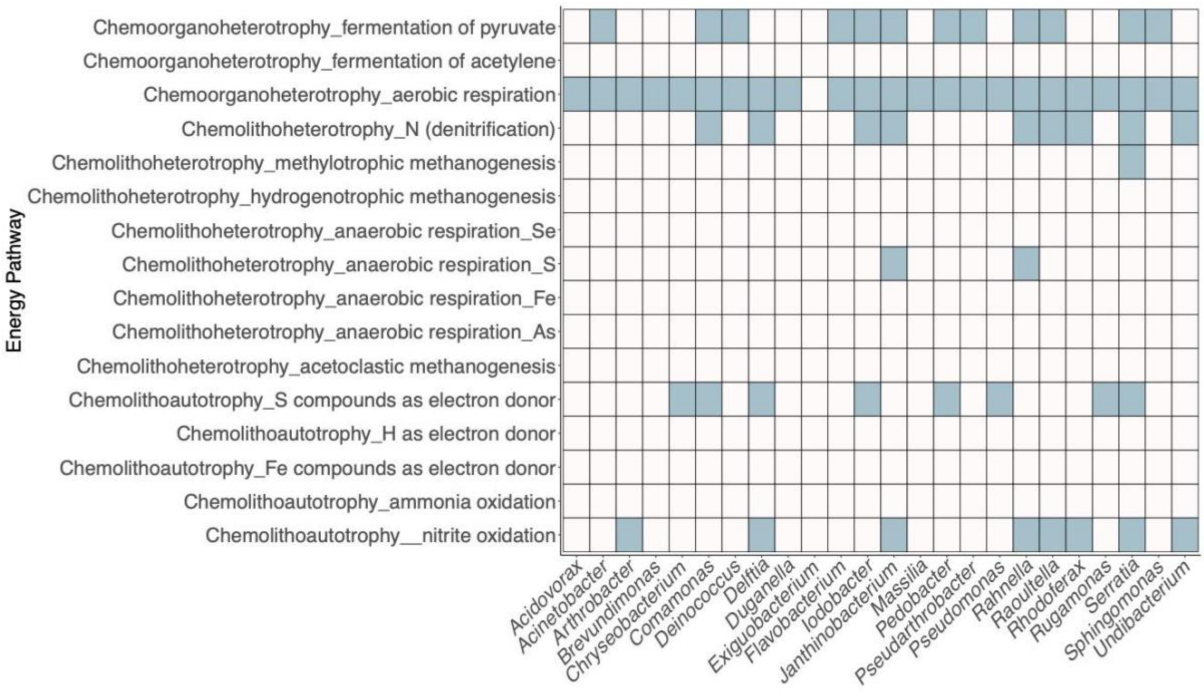

Supplementary Fig. 2: Energy pathways predicted from the microTrait pipeline. Colored tiles indicate the presence of a specific energy pathway in the bacterial isolates

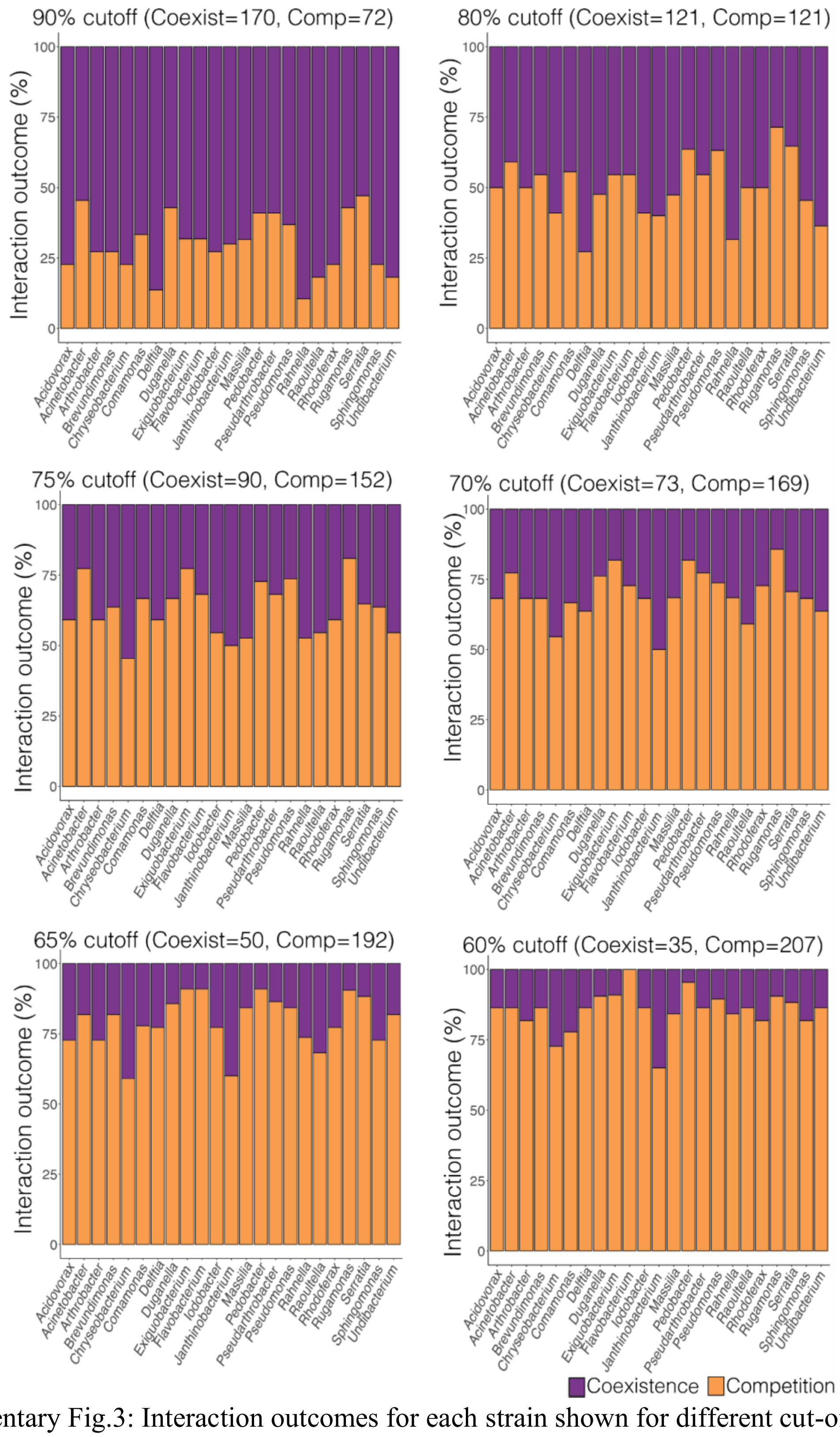

Supplementary Fig.3: Interaction outcomes for each strain shown for different cut-offs to define coexistence vs competition in the bacterial pairwise experiment. The cutoff values at the top reflect the percentage that determined the Competition outcome, meaning the interaction was defined as competitive if one of the strains had a percentage abundance equal or greater to the specified cutoff. The number of pairwise interactions categorized into either Coexistence (Coex) or Competition (Comp) is shown in parenthesis.

a)

Precursor mz	430.16
Molecular formula	C21H23N3O7
Adduct	[M + H]+
Confidence score	0.996
CSI:FingerID score	-60.692
Sirius Score	119.223
InChiKey	QSJOEQXFYCTBSY
Common name	Serratiochelin

b)

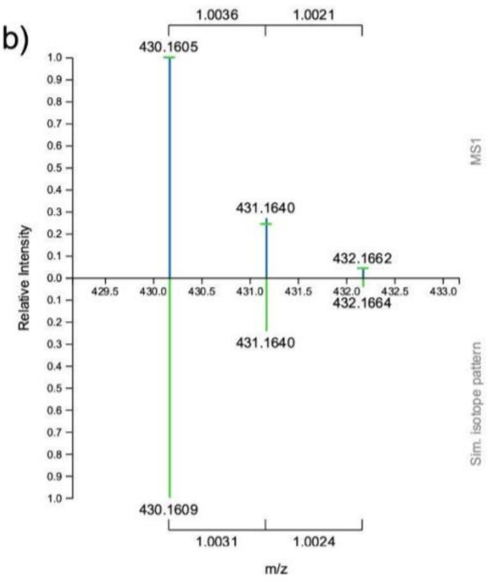

c) d)

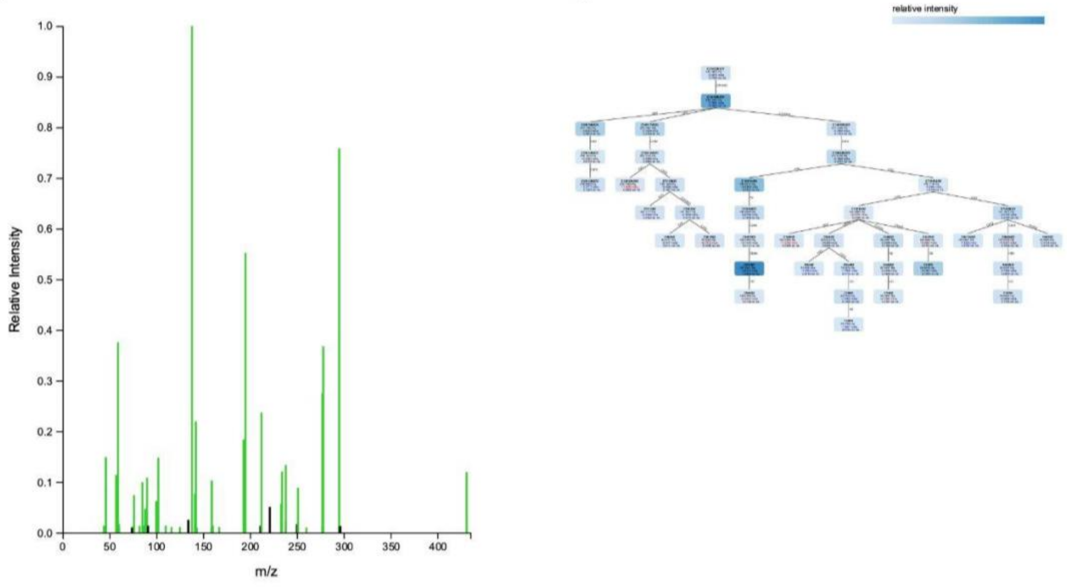

Supplementary Fig. 4: Compound identification of serratiochelin via MS/MS. (a) Summary table of compound identification parameters outputted by SIRIUS for the top hit. (b) MS1 mirror plot showing precursor isotope pattern of the observed molecule (above) versus a simulation of the theoretical molecule (below). (c) MS2 fragmentation spectrum of the compound. Green peaks represent matched ions, black peaks represent unmatched values. (d) Spectrum tree showing ions matched to MS/MS spectrum.

a)

Precursor mz	398.167
Molecular formula	C16H23N5O7
Adduct	[M + H]+
Confidence score	0.119
CSI:FingerID score	-266.196
Sirius Score	49.05
InChiKey	LXNCWECCTQQXPQ
Common name	Vanchrobactin

b)

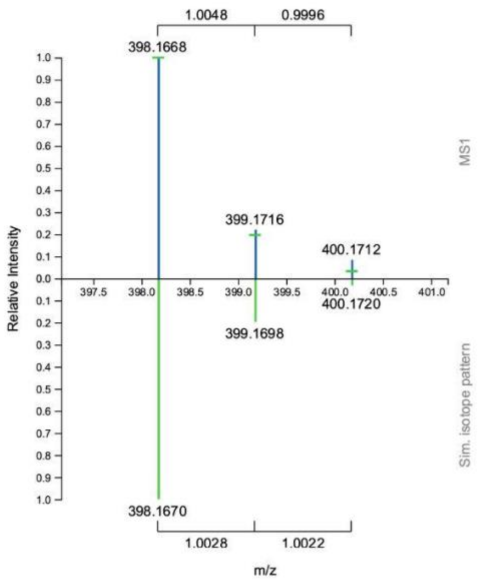

c)

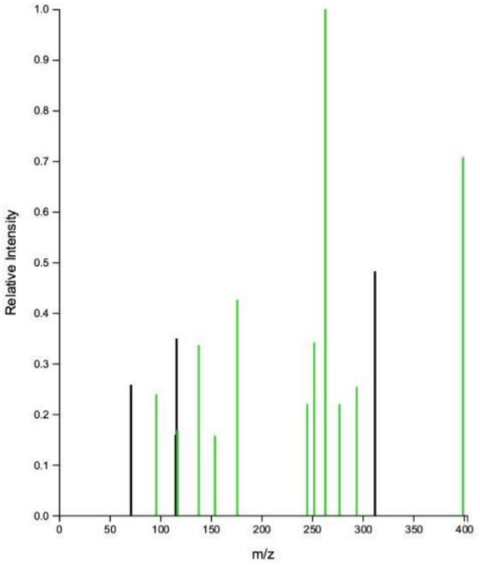

d)

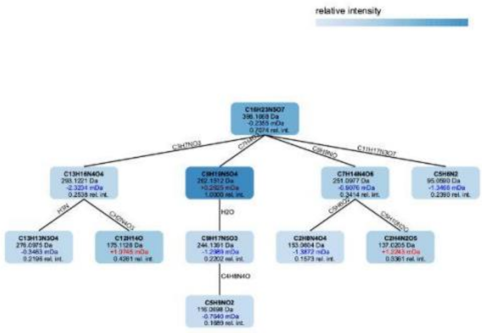

Supplementary Fig. 5: Compound identification of vanchrobactin via MS/MS. (a) Summary table of compound identification parameters outputted by SIRIUS for the top hit. (b) MS1 mirror plot showing precursor isotope pattern of the observed molecule (above) versus a simulation of the theoretical molecule (below). (c) MS2 fragmentation spectrum of the compound. Green peaks represent matched ions, black peaks represent unmatched values. (d) Spectrum tree showing ions matched to MS/MS spectrum.

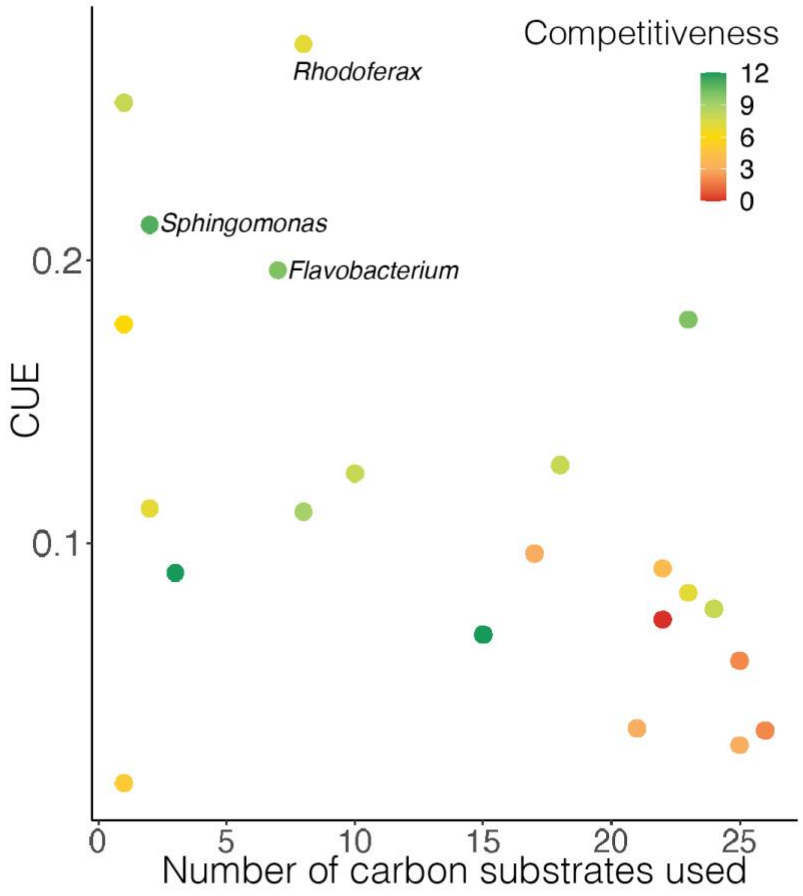

Supplementary Fig. 6: Correlation between carbon use efficiency (CUE) and the number of carbon substrates utilized by each strain in the Biolog Ecoplates (rho= -0.57, p=0.004). Colors indicate the competitive ability of isolates (number of other strains an isolate competitively excluded). Labels for *Rhodoferax*, *Flavobacterium and Sphingomonas* were added to highlight traits of some common GFS genera among our isolates.

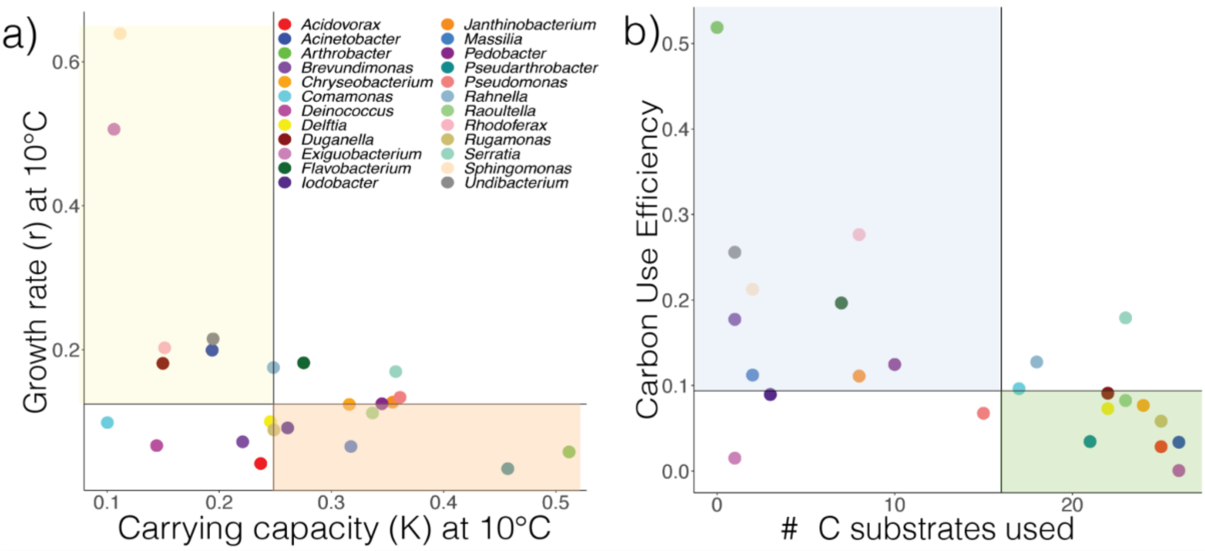

Supplementary Fig. 7: Trade-off between a) growth rate and carrying capacity of isolates at 10 °C, and b) between the number of carbon substrates they used and carbon use efficiency. Lines indicate the median values of each parameter. Rectangles in the upper and lower quadrants indicate isolate classified as r (yellow) and K (orange) strategists (b), or as oligotrophs (green) and copiotrophs (blue).

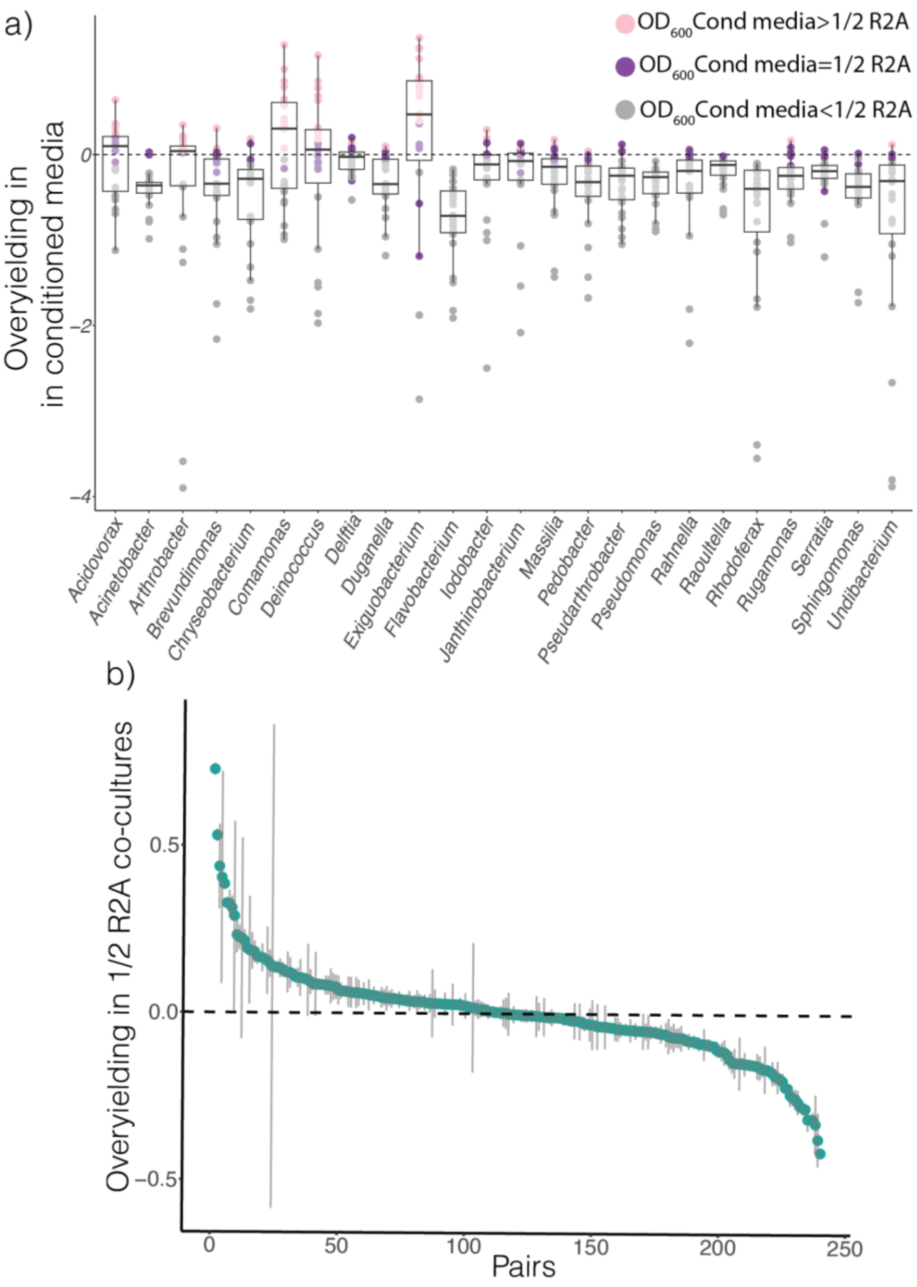

Supplementary Fig. 8: a) Overyielding in conditioned media defined as the log-ratio between the OD_{600} attained by the strains in conditioned media their OD_{600} in ½ R2A. Pink and purple circles represent instances of non-significant differences in growth between specific strains in conditioned media of others and in ½ R2A or greater growth in conditioned media respectively (i.e., the 95% CI includes the 0 line or is above it), while grey indicate that the strain grew significantly less in conditioned media. b) Instances of overyielding in ½ R2A co-cultures (cases in which when carrying capacity (K) of the pair was greater than the highest monoculture K in that pair).

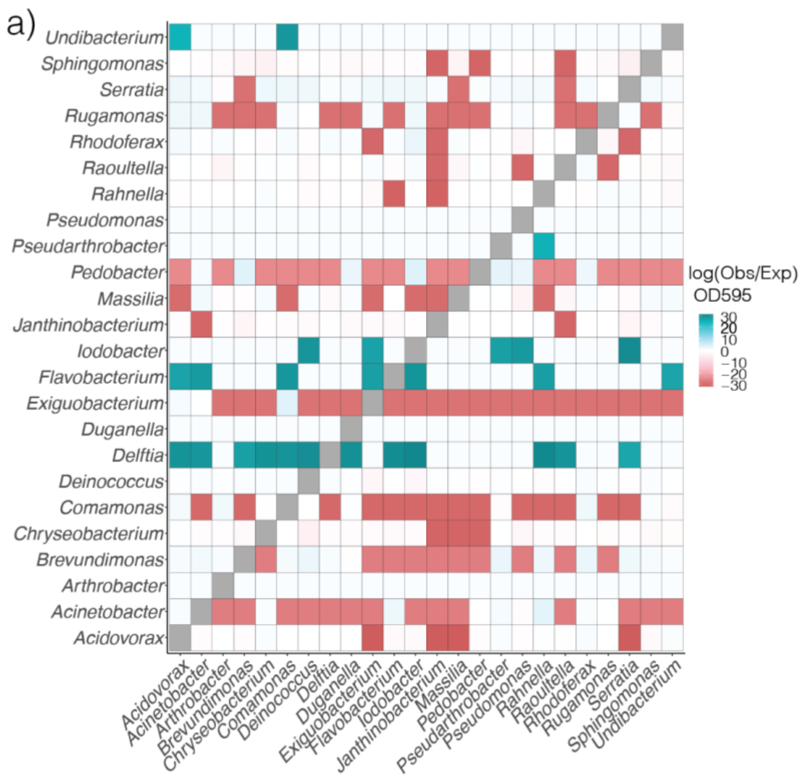

Supplementary Fig. 9: a) Heatmap of biofilm formation (OD_{590}) for each strain in conditioned media of all others (Obs) relative to each strain's biofilm formation in ½ R2A (Exp).

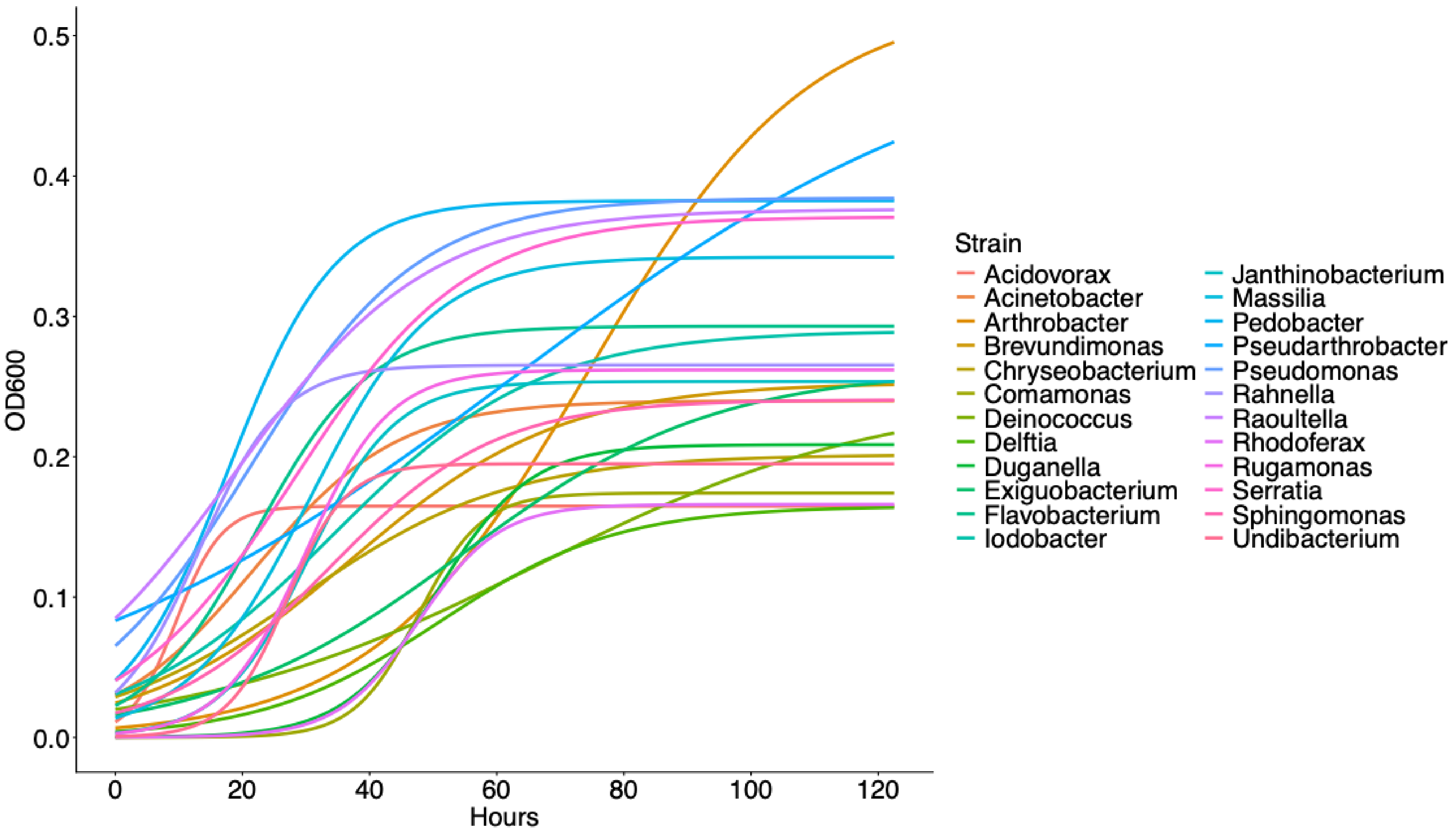

Supplementary Fig. 10: Logistic growth curves of the 24 strains growing in ½ R2A media at 10°C.
